# Enhancing liver disease diagnosis with hybrid SMOTE-ENN balanced machine learning models—an empirical analysis of Indian patient liver disease datasets

**DOI:** 10.3389/fmed.2025.1502749

**Published:** 2025-05-27

**Authors:** Ritu Rani, Garima Jaiswal, Nancy, Lipika, Shashi Bhushan, Fasee Ullah, Prabhishek Singh, Manoj Diwakar

**Affiliations:** ^1^Department of ECE, Bhagwan Parshuram Institute of Technology, New Delhi, India; ^2^School of Computer Science Engineering and Technology, Bennett University, Greater Noida, India; ^3^Department of Computer Science, Indira Gandhi Delhi Technical University for Women, New Delhi, India; ^4^Department of Computing, Universiti Teknologi PETRONAS, Seri Iskandar, Malaysia; ^5^Department of Computer Science Engineering, Graphic Era Deemed to be University, Dehradun, Uttarakhand, India; ^6^Department of CSE, Graphic Era Hill University, Dehradun, Uttarakhand, India

**Keywords:** imbalanced data, SMOTE, SMOTE-ENN, SMOTE-Tomek, logistic regression, SVM, random forest, KNN

## Abstract

**Introduction:**

The liver is one of the vital organs of human body that performs some of the most crucial biological processes such as protein and biochemical synthesis, which is required for digestion and cleansing. A large number of patients are suffering from liver disease and hence it has become a life-threatening issue around the world. Annually, around 2 million people die because of liver disease, this accounts for around 4% of all deaths, due to factors like obesity, undiagnosed hepatitis, and excessive alcohol consumption. These factors accumulate and deteriorate the liver condition. Immediate action is necessary for timely diagnosis of the ailment before irreversible damage is done.

**Methods:**

The work aims to evaluate some of the traditional and prominent machine learning algorithms, namely, Logistic Regression, K-Nearest Neighbor, Support Vector Machine, Gaussian Naïve Bayes, Decision Tree, Random Forest, AdaBoost, Extreme Gradient Boosting, and Light GBM for diagnosing and predicting chronic liver disease. Also, real-world datasets often have imbalanced class distributions, causing classifiers to perform poorly, leading to low accuracy, precision, recall values and high misclassification. The Indian Patient Liver Disease (ILPD) datasets also face an imbalance issue. This work presents two hybrid models, namely SMOTEENN-KNN and SMOTEENN-AdaBoost, which can robustly handle the problem of imbalance in real-world datasets, in addition to improving the accuracy of liver disease prediction. We have also designed a hybrid model which involves the combination of Recursive Feature Elimination (RFE) for feature selection, SMOTE-ENN to tackle the problem of data imbalance and Ensemble learning for enhanced predictions.

**Results:**

The research work also proposed Hybrid Ensemble model on the ILPD and BUPA Liver Disorder Dataset. For the ILPD dataset, the model achieves an overall accuracy of 93.2% whereas for the BUPA dataset, the model attains an accuracy of 95.4%. The Brier Score loss for ILPD dataset is 0.032 and 0.031 for the BUPA Liver Disorder Dataset.

**Discussion:**

The research work highlights the potential of data balancing techniques and Ensemble models to improve predictive accuracy in liver disease diagnosis.

## 1 Introduction

**Figure 1:**
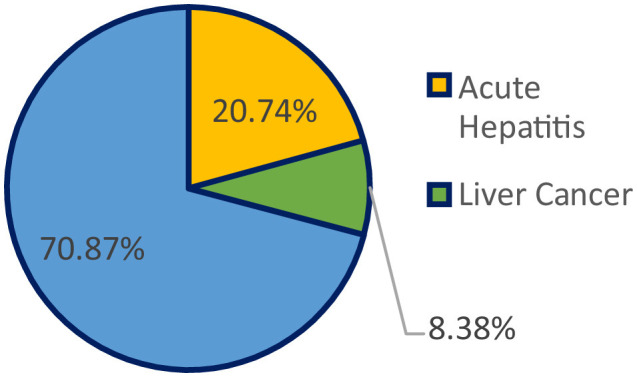
Percentage of deaths due to multiple liver diseases.

Every year, liver disease causes 2 million fatalities, or 1 out of every 25 deaths around the world, accounting for 4% of all deaths (1). Liver cancer, the 16th most frequent cause of death worldwide, and cirrhosis, currently the 11th most frequent cause of death worldwide, account for 3.5% of all fatalities worldwide, with cirrhosis-related complications, viral hepatitis (2), and hepatocellular cancer causing one million cases each (3). Cirrhosis is a disorder that harms the liver by scarring and inflicting damage; the serious side effects that can result from it include liver failure, hemorrhage, infections, and cancer. Alcohol misuse, viral hepatitis, fatty liver disease (4), autoimmune illnesses, and hereditary conditions are only a few of the causes of cirrhosis. Figure 1 shows the percentage of deaths due to multiple liver diseases.

The liver, a wedge-shaped organ, is the second-largest organ in the human body, after the skin, and is located in the upper right abdominal cavity. It is also the body's largest gland, secreting hormone-like substances. The liver performs more than 500 functions in the human body, and it maintains most of the organs that are essential to life. We used the Indian Patient Liver Disease dataset for this study. The dataset we select is unbalanced, to evaluate real-world performance, as actual data is normally unbalanced. The imbalance class must be handled prior to obtaining the dataset and training any model that implements a machine learning (ML) algorithm since the classification methods assume that the majority and minority classes are balanced.

### 1.1 Challenges in liver disease detection

- Many people do not experience many symptoms until their illness has progressed, at which point the medication may be useless.
- Due to the limited awareness about liver disease and its symptoms.
- Due to the scarcity of centers that have proper clinical experience and provide the necessary laboratory skills to diagnose liver disease.
- Liver disease is often overlooked in primary care, and liver function tests (LFTs) are frequently ordered in basic care to assess liver health.

To detect liver illness early, machine learning algorithms can be used to reduce the risk of complications, ultimately enhancing the patient survival rates and minimizing subsequent issues.

### 1.2 Problem with the traditional method in detecting liver disease

The conventional techniques for identifying liver disease include tests for liver function, which are specific blood examinations used to detect liver disease. The additional blood tests may be used to check specific genetic or hepatic issues. Imaging examinations, including magnetic resonance imaging (MRI), computed tomography (CT) scan, and ultrasound, are utilized to demonstrate liver damage. To evaluate for liver disease, a biopsy of the liver can be performed. A liver biopsy is an intrusive procedure that can end in hemorrhage, biliary peritonitis, and pneumothorax, among other problems. CT scan, MRI, and ultrasound are some imaging tests that can show liver damage; however, however, these tests are expensive. Non-invasive techniques, including clinical prediction scores, elastography, and ultrasonography, have been used as alternatives to liver biopsies.

In the traditional approach, the patient had to consult a doctor to undergo certain liver function tests, blood tests, imaging tests, such as MRI and CT scans, and have a tissue sample checked. Then the doctor integrates qualitative and quantitative information from the multiple tests to diagnose and recommend the treatment. In recent years, artificial intelligence and machine learning techniques have emerged and advanced the extraction process of relevant information from vast and complex clinical datasets. Additionally, researchers studying in this domain have shown that various machine learning models or hybrid models (5) can be relevant for predicting the risk of liver disease. These algorithms can help detect early liver disease, which, in turn, helps to initiate treatment in the early stages of the disease.

However, the majority of real-world datasets are imbalanced. The imbalanced dataset presents various problems while predicting liver patients. Therefore, the imbalanced dataset must be addressed to achieve more accurate results. The Indian Patient Liver Disease (ILPD) dataset has a significantly imbalanced class distribution, where the majority class (liver disease) is substantially more prevalent than the minority class (no liver disease). Traditional ML models, such as logistic regression, decision trees, or support vector machine (SVM), tend to favor the majority class, leading to poor performance in predicting the minority class. This may lead to low recall and F1-score for the minority class (non-liver disease patients) as well as poor generalization to unseen data, particularly for minority cases.

The ILPD dataset contains noise in features, such as outliers in enzyme levels or misclassified labels. Due to this, many traditional ML models, such as K-nearest neighbors (KNN) and logistic regression, are sensitive to noise, leading to biased predictions and reduced accuracy.

Traditional models are often optimized for accuracy, which is not suitable for imbalanced datasets, as accuracy can be misleading, as it may reflect correct predictions for the majority class while ignoring poor performance on the minority class.

### 1.3 Problem with imbalanced datasets

An imbalanced dataset is any classification dataset with uneven class proportions. It can be problematic if not addressed properly. The following problems are associated with machine learning algorithms that use classification and are applied to imbalanced datasets:

- These classification algorithms perform better for the majority class and worse for the minority class.
- Thus, there exists a bias toward the majority class, and the algorithm ultimately overlooks the minority class.
- The minority class is often poorly classified, resulting in a high misclassification rate.
- Misleading accuracy score and suboptimal model performance.

### 1.4 Major contributions of the study

- A comprehensive review of the relevant literature existing in this domain has been discussed.
- An extensive review of the ML models that we have implemented, as well as all the balancing techniques used, is discussed.
- The implemented traditional ML models, as well as hybrid models incorporating all the balancing techniques mentioned earlier in this article, are discussed.
- Next, a comparative analysis of all implemented traditional ML models and hybrid models is given.
- After that, the hybrid ML models, Synthetic Minority Oversampling Technique-Edited Nearest Neighbors (SMOTE-ENN)–KNN and SMOTE-ENN-AdaBoost, are suggested, which are quite effective in liver disease detection as well as for imbalanced datasets.
- A hybrid model, which involves the combination of Recursive Feature Elimination (RFE) for feature selection, SMOTE-ENN to address the problem of data imbalance, and ensemble learning, is implemented and analyzed on the ILPD dataset. This hybrid ensemble model outperforms other state-of-the-art studies and achieves an accuracy score of 93.2%, Brier score loss of 0.032.
- The proposed hybrid ensemble model is also implemented on the BUPA Liver Disorders Dataset, and comparable results with the ILPD datasets are achieved to ensure the generalizability of the proposed model.

### 1.5 Organization of the article

Section 1, “Introduction,” discusses the liver disease statistics, challenges in liver disease detection, and problems with imbalanced datasets. Section 2, “Literature Review,” elaborates on the previous research study in this domain by numerous researchers. Section 3, “Methodology and Implementation,” details the workflow of the complete system of liver disease prediction that we have used, and describes the implemented models and the balancing techniques employed. Section 4, “Results and Analysis,” discusses the results obtained from implementing traditional models and hybrid models. In this study, we have conducted a comparative analysis of the implemented models based on evaluation metrics, such as recall, precision, accuracy, F1-score, and receiver operating characteristic (ROC) curve–area under the curve (AUC) scores. Section 5, “Conclusion,” summarizes the key points discussed in this article and talks about the importance and future scope of the research.

## 2 Literature review

A considerable amount of research is being carried out on liver disease prediction, which is of paramount importance in today's scenario, and is discussed in Table 1. Supervised and unsupervised machine learning models and algorithms for the prediction of liver disease risk are studied, and a comparative analysis is performed (6, 7). Among the most influential studies in ML with reference to this topic can be attributed to Mondal et al. (8) used different classification algorithms, including logistic regression, KNN, and SVM for liver disease prediction. The comparative analysis of the aforementioned algorithms has been compiled, on the basis of accuracy, calculated using a confusion matrix. The KNN model shows an accuracy of 73.97%, the logistic regression model has an accuracy of 73.97%, and the SVM model has an accuracy of 71.97%.

**Table 1:** Previous studies in liver disease detection.

**Author**	**Dataset employed**	**Methodology**	**Preprocessing**	**Classification algorithm**	**Balancing techniques used**	**Evaluation metrics**	**Result**	**Nutshell of the article**
Singh et al. (9)	IPLD	1. Data selection 2. Preprocessing 3. Feature selection 4. Data transformation 5. Model selection 6. Evaluation	Data cleaning: filling missing values; transforming nominal attributes to binary attributes	Logistic regression (LR), SVM, and KNN	None	Confusion on matrix, specificity, and sensitivity	LR: 73.97% KNN: 73.97% SVM: 71.79%	This study compares LR, KNN, and SVM for liver disease prediction. Accuracy scores and a confusion matrix are used to compare algorithms. LR and KNN have the highest accuracy, indicating their suitability for liver disease prediction.
Keerthana et al. (10)	ILPD	Data selection, data preprocessing, model implementation, and result analysis	Handling null values and duplicate values	Logistic regression	None	Confusion on matrix, ROC score, and ROC curve	LR: 85.96%	This study uses machine learning's logistic regression method for the detection of liver disease, suggesting that further improvement in accuracy can be obtained by decision trees and KNN algorithm.
Rakshith et al. (12)	ILPD	1. Building and raining the system [Dataset -> Feature selection -> Classification algorithm -> build model] 2. Testing the model Entering the details and prediction	Handling null values, duplicate values, and missing values	KNN, SVM, Naïve Bayes (NB), and ANN	None	Accuracy, confusion matrix	SVM: 100% KNN: 70% NB: 55.56% ANN: 99.9%	This study predicts liver disease risk using ML techniques. The system uses a trained model for predicting the risk of liver disease. The most accurate model, SVM, achieved 100% accuracy on a dataset, indicating that it can predict liver disease risk with 90% or more accuracy.
Arbain et al. (13)	ILPD	1. Data collection/selection 2. Data preprocessing 3. Implement the decision tree algorithm 4. Evaluation compare performance	Data cleaning (locates and fixes errors or discrepancies in the data). Waikato environment for knowledge analysis (WEKA), a data mining tool, is used.	LMT, J48 (22), random tree (RT), RF, REPTree, decision stump, and Hoeffding Tree	None	Accuracy, mean absolute error (MAE), Precision, Recall, F-score, Kappa statistics, and runtime	Decision stump, RT, Hoeffding, and LMT Tree have the accuracy rates of 70.67%, 69.47%, 69.75%, and 69.30%. J48 has the worst accuracy of 65.69%.	This study compares decision tree algorithms for the diagnosis of liver disease, assessing various techniques and finding the most relevant one. Decision stump has the highest accuracy of 70.67%.
Fernando et al. (14)	ILPD	The dataset is partitioned into testing and training sets, with each model trained and tested.	The dataset is converted using an argument parser to convert class label information into numbers.	Random forest (RF), multilayer perceptron (MLP), KNN, and SVM	None	Accuracy	Accuracy: RF: 69% KNN: 67% SVM: 74% MLP: 68% The accuracy reports for all ML approaches are similar.	The ILPD was analyzed using four ML approaches: RF, SVM, KNN, and MLP. The SVM approach was found to be the best-fitting model, outperforming all others. Comparative analysis highlighting the importance of understanding prediction and model performance.
Mostafa et al. (16)	Data collected (24) from the University of California Irvine Repository.	1. Data collection 2. Data Cleaning and Preprocessing 3. Risk factor determination 4. Classification 4. Trained model 5. Evaluation	•Data visualization •Imputing missing values using MICE •PCA to minimize dimensionality.	RF, SVM, and ANN	SMOTE	Accuracy, precision, sensitivity, F-1-score, specificity, and ROC analysis	RF had the highest sensitivity (0.9904) and accuracy (98.14%), while SVM performed better in terms of running time. ANN showed poor performance.	The study demonstrates that ML algorithms can detect liver disease in blood donors with high accuracy using correlations with risk factors. SVM and RF outperform ANN. AUC–ROC and cross-validation pair *t*-tests confirm the result.
Nahar and Ara (17)	ILPD	1. Dataset selection 2. Preprocessing 3. Data transformation 4. Data mining 5. Interpretation and evaluation 6. Knowledge discovery	•Data visualization •Handling missing values and detecting the possible outliers.	Random forest (RF), KNN, logistic regression (LR), and auto neural (AaN)	Random Sampling	Accuracy, ROC train chart, and index	Accuracy: KNN: 99.794% 0.845 ROC train chart. Auto neural: 99%, LR with backward selection: 99.764%	This study used four classification algorithms to predict liver disease. RF provided the best classification results, but it was overfitting and thus making it unsuitable. KNN outperforms all the implemented algorithms.
Veeranki and Varshney (19)	Behavioral risk factor surveillance system (BRFSS) 2015; Heart Disea se Health Indicators Dataset	1. Data selection 2. Data preprocessing 3. Balancing techniques 4. Model implementation 5. Result analysis	Dealing with missing values and null values	KNN, Gaussian NB, decision tree (DT) XGBoost, light gradient boosting (LGB) machine, AdaBoost, and random forest (RF)	ROS, SMOTE ADASYN, random undersampling (RUS) SMOTE-Tomek, and SMOTE-ENN	Accuracy, precision, sensitivity, Matthews correlation coefficient (MCC) score, F1-score, specificity, and ROC analysis	RF has high accuracy (0.90) for all sampling method except RUS. XGBoost, LGB, and AdaBoost showed above 0.92 performance for the cluster centroid algorithm.	Random forest has the highest accuracy among the sampling techniques used, except the RUS method. SMOTE-Tomek, SMOTE-ENN hybridrandom sampling methods, and cluster centroid method have higher accuracies as compared to 0.90 for DT, XGBoost, LGBM, AdaBoost, and RF. Tomek link algorithm has the lowest MCC value.
Gupta et al. (23)	ILPD	1. Data collection 2. EDA 3. Preprocessing 4. Feature selection 5. Classification using ML 6. Performance evaluation	•Imputation of •missing values •Dummy encoding •Elimination of •duplicate values •Outlier detection, and elimination •Resampling	Logistic regression (LR), decision tree (DT), KNN, RF, gradient boosting (GB), XGBoost, light GBM	SMOTE	Deviance, Akaike information criterion (AIC), pseudo R2, accuracy, ROC, precision, AUC, recall, specificity, F1-score, kappa statistic, cross-entropy	LR: 57%, NB: 54% DT: 61 RF: 63 XGBoost: 60% AdaBoost: 62% LGBM: 63% KNN: 57%	This study examines ML algorithms for predicting liver disease. RF, LGBM, and AdaBoost algorithm provided better accuracy than other classification algorithms, indicating that light GBM is suitable for predicting liver disease.
Kumar et al. (25)	BCD, ILPD, CKD, CHD, and Pima Indians Diabetes	1. Data collection/selection 2. Data preprocessing 3. Balancing techniques 4. Implement ML algorithm 5. Evaluation 6. Compare performance	Data cleaning, data transformation, and missing value imputation	LR, KNN, DT, SVM, and artificial neural network (ANN)	Undersampling, random over sampling, SMOTE SVM-SMOTE ADAS YN, SMOTE-ENN, and SMOTE-Tomek	Confusion matrix, accuracy, recall, precision, F1-score	SMOTE-ENN + KNN model gives the best result for all datasets with an accuracy of more than 90%. SMOTE with LR has the highest accuracy of 99.2%, over the CHD dataset.	The study evaluates six classifiers with respect to seven class balancing techniques on five imbalanced clinical datasets. SMOTE-ENN balancing method outperforms all other methods, with KNN providing the highest precision, accuracy, F1-score, and recall. KNN–SMOTE-ENN is most suitable for detecting liver disease, diabetes, and coronary heart disease.
Kumar et al. (26)	ILPD	1. Data collection 2. Preprocessing 3. Data visualization 4. Model implementation 5. Evaluation metrics result analysis	•Data cleaning •Data visualization	Logistic regression (LR), KNN, DT, SVM, Naïve Bayes (NB), and RF	None	Accuracy, precision score, recall score, F1-score, and specificity	•Accuracy: LR: 75% NB: 53%•Precision: LR: 91% NB: 36%•Sensitivity: SVM: 88% KNN: 76%•F1-score: LR: 83% and NB: 53%. •Specificity: DT: 48% LR: 47%.	This study evaluates the performance of six ML algorithms (LR, KNN, RF, DT, SVM, and NB) in predicting chronic liver disease, and finding high accuracy rates. The LR classification technique is found to be more effective than other classifiers.

Singh et al. (9) incorporate the detection of the presence of liver disease using the logistic regression algorithm. The preprocessing methodologies, such as the removal of duplicate values, null values, dealing with categorical data using encoding methods, and scaling features, have been used. The dataset is partitioned 80% for training the model and 20% for testing the model. The **final accuracy score** achieved in this model is **0.859649**. It proposes to improve the accuracy further.

Keerthana et al. (10) use various ML models for predicting liver disease. They have designed a system in which the patient will have to submit the report of the blood tests performed, after which the system employs the model that is most accurate for predicting if liver disease is present in the patient. The Python^1^ programming language has been used, and the Sklearn library was instrumental in the construction of the machine learning models, which use SVM, Naïve Bayes, KNN, and artificial neural network (ANN). They found that SVM provided the most accurate result (11). Rakshith et al. (12) studied various ML models, including logistic regression, KNN, decision tree, random forest, gradient boost, XGBoost, and light GBM, concluding that the LGBM algorithm would deliver the maximum accuracy for predicting liver disease. They balanced their dataset using SMOTE and various classifiers, base, and advanced algorithms. Random forest, AdaBoost, and LGBM models give better accuracy than other classification models.

Arbain et al. (13) investigated data mining algorithms for the prediction of liver disease using the technique of random sampling of imbalanced data. RF, despite providing the best classification results, is overfitted due to insufficient data. KNN outperforms other algorithms, such as LR, auto neural, and RF, with an accuracy rate of 99.794%, and a 0.845 ROC train chart. Fernando et al. (14) found that the ensemble classifiers, such as random forest, AdaBoost, and XGBoost, outperform base classifiers most likely due to their inherent behavior of the ensemble principle (15). Decision tree and KNN classifiers showed good performance, and the Gaussian Naïve Bayes classifier showed the least performance. Random forest algorithm showed an accuracy of above 90% in all sampling techniques. The hybrid methods SMOTE-Tomek and SMOTE-ENN performed best. Mostafa et al. (16) compared binary classifier machine learning algorithms, RF, SVM, and ANN. Machine learning techniques enhance inference-based diagnosis by incorporating risk factors for predicting liver disease. RF and SVM showed better performance than ANN, but SMOTE oversampled the minority group. The study suggests using multinomial classification instead of binary classification to compare performance and understand interpretability.

Nahar et al. (17) compared numerous decision tree algorithms (18) [e.g., logistic model tree (LMT), random tree, J48, random forest, REPTree, decision stump, and Hoeffding Tree] to diagnose liver disease and tried to find the model that performed best in the decision tree classification. The results reveal that decision stump has an accuracy of 70.67%, which is the highest among all the implemented algorithms. Veeranki and Varshney (19) focused on classifying the genetic data of liver patients from those without liver disease. The study uses four ML techniques: random forest (RF), multilayer perceptron (MLP) model (20), KNN, and SVM. The dataset is converted using an argument parser. The results show that the SVM approach secures 74% accuracy, RF: 69%, KNN: 69%, and MLP: 68%. They conclude that the SVM approach is the best fit model for liver disease prediction (21). Rahman et al. (22) evaluated the comparative performance of six ML models (LR, KNN, DT, RF, SVM, and NB) in the prediction of liver disease. The accuracy of the mentioned techniques was assessed using various techniques for measurement. The results revealed that LR obtained the maximum accuracy at 75%, while KNN, DT, RF, SVM, and NB achieved the lowest accuracy at 53%.

The other most influential study is ascribed to Gupta et al. (23). They used seven balancing techniques, namely under-sampling, various techniques, including random oversampling, Adaptive Synthetic Sampling (ADASYN), SMOTE, SMOT-ENN, SMOTE-Tomek, and SVM-SMOTE, were implemented along with six disease prediction models: logistic regression, SVM, decision tree, KNN, and ANN. These models were evaluated on five different prominent clinical datasets, namely, the Pima Indians Diabetes Database, ILPD, bacterial cell death (BCD), congenital heart disease (CHD), and chronic kidney disease (CKD). Notably, KNN–SMOTE-ENN achieved the maximum accuracy, precision, recall, and F1-score across the various ML techniques, outperforming other approaches on the BCD dataset.

## 3 Methodology and implementation

### 3.1 Dataset description

There are 167 records of non-liver patients and 416 records of liver patients in the Indian Liver Patient Dataset (ILPD). The information was gathered in northeastern Andhra Pradesh, India. Patients are classified as either liver patients or not by the class label. The dataset includes 142 records of female patients and 441 records of male patients. On the basis of the presence of various chemical compounds in the human body (bilirubin, proteins, albumin, and alkaline phosphatase [Alkphos]) as well as tests such as SGPT (alanine aminotransferase or ALT), (GOT (aspartate aminotransferase or AST), it is possible to predict if the person is a patient, that is, whether he or she has to be diagnosed. Age, sex, total bilirubin, direct bilirubin, alkaline phosphatase, AST, ALT, albumin, globulin ratio (A/G), and total proteins are the 11 dataset attributes (ILPD).

The “Albumin_and_Globulin_Ratio” has null values, for which we have taken the mean of this attribute and replaced all null values with this mean. We checked for duplicate values. Since there is no unique patient identifier against each observation, and since it is highly improbable that two subjects have the same exact feature values, we can conclude that the records are possibly duplicates.

**Figure 2:**
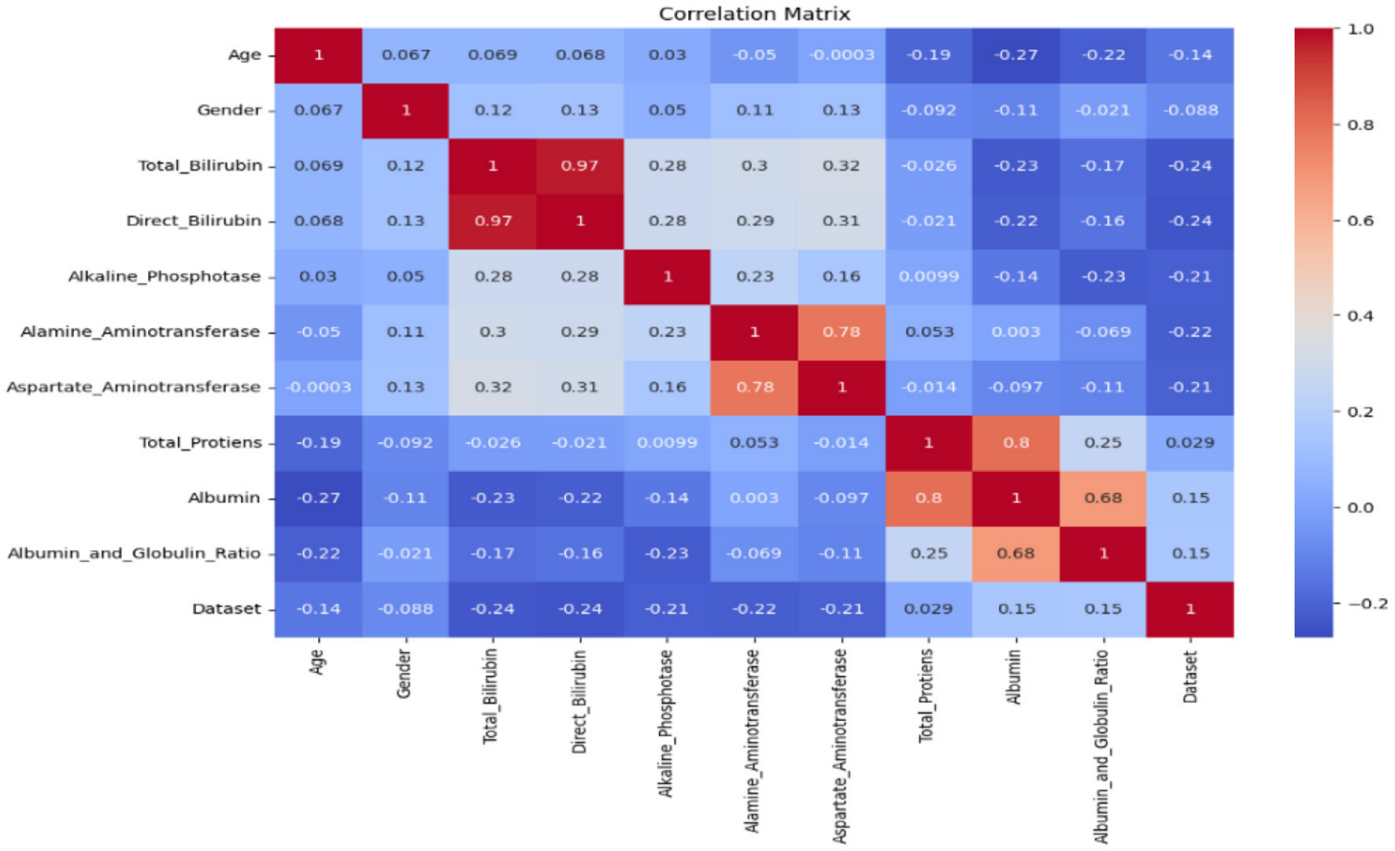
Correlation matrix.

The correlation metrics shown in Figure 2 is used to summarize a large dataset and to find the correlation among different features of the dataset. The matrix has rows and columns. Every cell in the matrix shows correlation coefficient. The coefficient value of 1 indicates strong correlation, 0 represents a neutral relation. The correlation matrix allowed us to identify features that were strongly correlated with the target variable (liver disease diagnosis) and prioritize them for inclusion. It helps to detect multicollinearity among independent variables. For example, features such as total bilirubin and direct bilirubin showed a very high correlation (r > 0.9), and we retained only one of them to avoid redundancy. It helps remove features with negligible correlation to the target variable (correlation coefficient < 0.1); for example, age, total proteins, etc. are unlikely to contribute significantly to the prediction task.

We selected relevant features using these criteria to ensure that the input data provided meaningful information for the machine learning models while avoiding overfitting caused by redundant features. The correlation matrix is used to get a better understanding of the dataset as it makes visualization easier.

### 3.2 Traditional methodology

**Figure 3:**
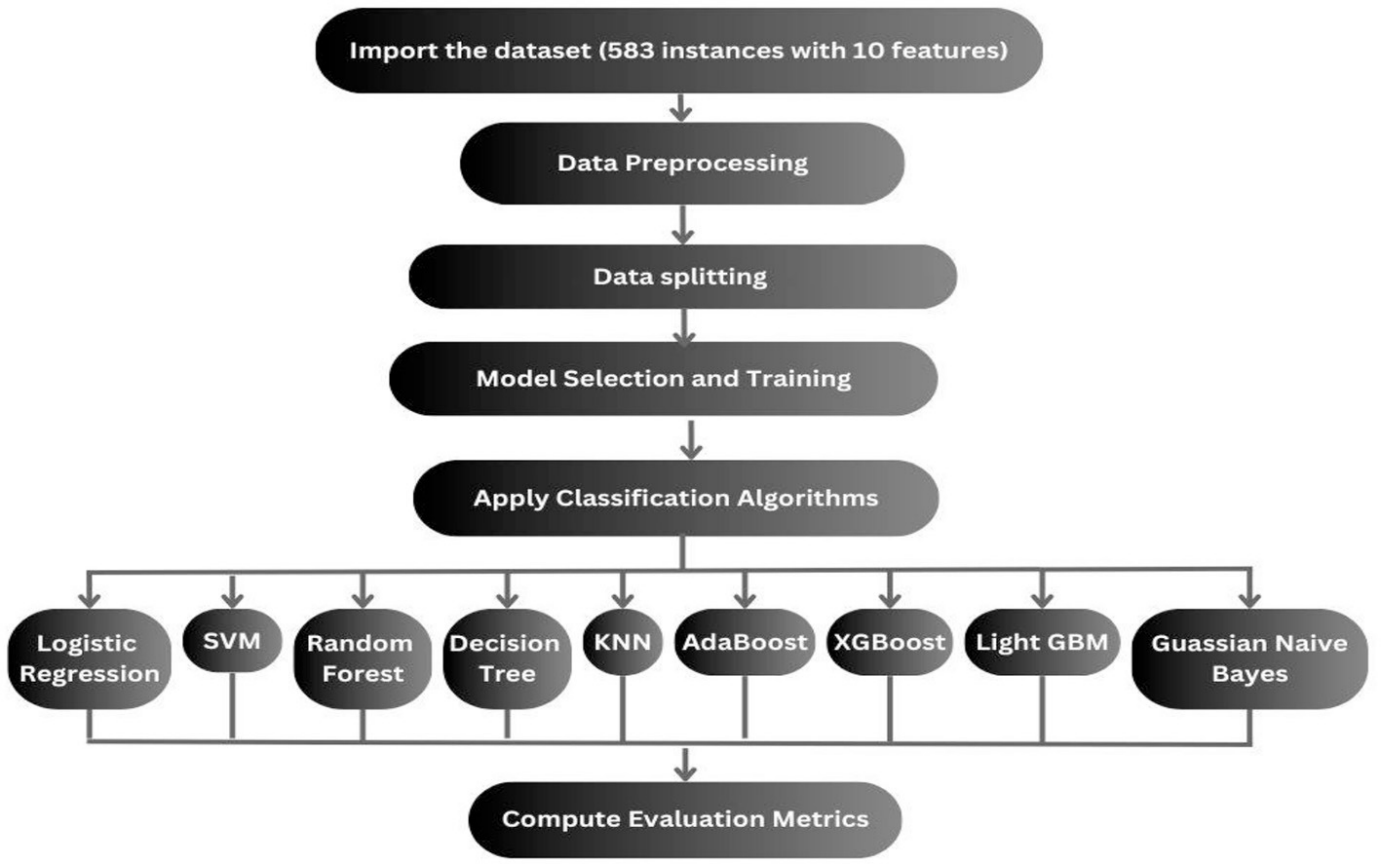
Workflow of the previous followed methodology.

The traditional methodology to design any ML model for the prediction of liver disease is displayed in Figure 3 includes the following steps. First, the dataset is fetched, and data preprocessing is performed, which is then followed by data visualization. Then the partition of the dataset into train and test data is done, after which the model is trained on the training data, and the trained model is now tested on the test data. Finally, evaluation of the implemented model is performed (27, 28).

We employed grid search with 5-fold cross-validation for all classifiers to identify the optimal set of hyperparameters. The dataset was divided into 5 folds, with four used for training and one for validation in each iteration. This process was repeated for each fold, and the average performance across folds determined the best hyperparameter combination.

### 3.3 Proposed methodology and ML models

**Figure 4:**
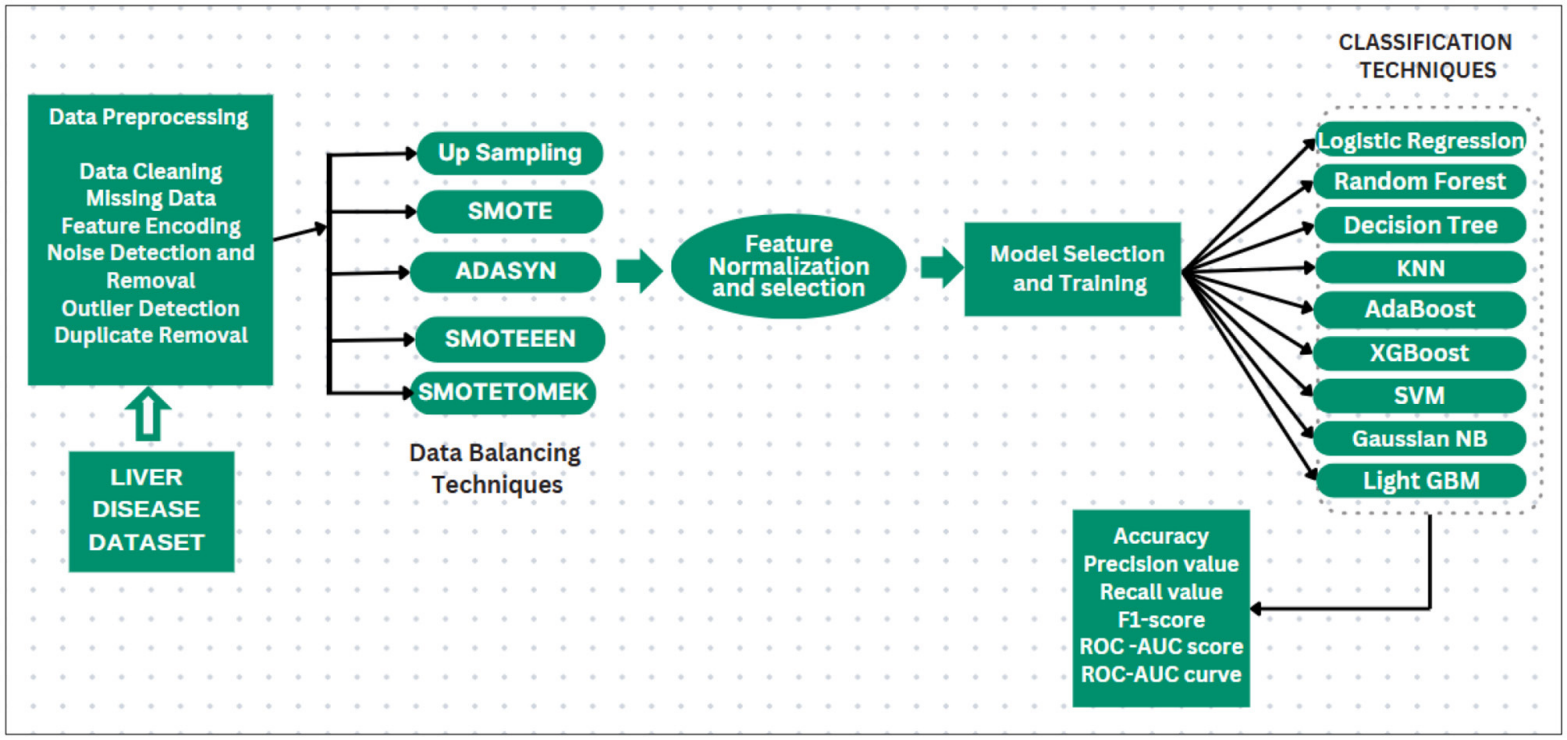
Prediction system for liver disease dataset.

The dataset we select is imbalanced. If the models are implemented without handling the imbalanced dataset, then the implemented models will be biased toward the majority, and some models may be overfitted. First, it is needed to balance the dataset. Multiple balancing techniques are used on 9 classification techniques. The balancing techniques and classification techniques are mentioned in the following subsection, and the proposed methodology is displayed in Figure 4.

i. **Dataset collection**: selecting or collecting the data of liver patients for selecting meaningful records to obtain and analyze the productive information by performing various data mining techniques. The dataset contains both numeric and categorical features such as age, sex, liver function tests (e.g., bilirubin, SGOT, SGPT, etc.), and the target variable indicating whether the patient has liver disease (Label: 1 for liver disease, 2 for no disease).
ii. **Data preprocessing**: it is a critical phase in the data mining process. It includes the cleaning as well as processing of the raw data so that it can be analyzed. Some common data preparation steps are listed as follows:

- **Data cleaning:** the data might have some missing and irrelevant parts. Data cleaning is performed to handle such discrepancies in data.
- **Missing data**: when some data is missing in the dataset, then those values must be handled either by ignoring the tuples or filling in the missing values. We addressed missing data during preprocessing by removing rows with missing values and imputing missing values using the mean or median (depending on the feature type) to prevent bias during model training. We used isnull() to find missing values, and then we calculated each attribute's percentage of null values. After that, we filled in the missing values by the particular attribute's mean and median of available values.
- **Feature encoding**: categorical features, such as sex, were encoded into numerical values (e.g., male = 1; female = 2) to ensure they could be processed by machine learning algorithms.
- **Noise detection and removal:** prior to applying balancing techniques, noise in the dataset was detected and removed. This involves:

- **Outlier detection**: identifying and handling outliers using the Z-score method. Outliers that may distort model training were either removed or capped.
- **Duplicate removal:** identifying and removing duplicate records in the dataset to avoid biased model learning. Handling noise at this stage helps ensure that the dataset used for balancing and training the models is of higher quality, ultimately leading to more robust model predictions.

**iii. Balancing the imbalanced dataset:** since the dataset suffers from class imbalance, data balancing techniques were applied to generate synthetic samples for the minority class.
**iv. Feature normalization:** After applying the balancing techniques, the features were normalized using the StandardScaler from scikit-learn.^2^ This step ensures that each feature has a mean of 0 and a standard deviation of 1, which is crucial for algorithms sensitive to feature scaling (e.g., KNN, logistic regression, SVM).
**v. Feature selection:** The initial set of features, including age, sex, various liver function test results (e.g., bilirubin, SGPT, SGOT, etc.), was used for training the models. A heatmap of feature correlations was generated to assess the importance of each feature. The features that were highly correlated with each other were considered for removal to reduce redundancy and improve model performance. First, the correlation matrix is calculated for all features in the dataset. This matrix quantifies the pair-wise relationships between all features using the Pearson's correlation coefficient (for linear relationships) and Spearman's rank correlation coefficient (for non-linear relationships). Then, a threshold value is set to determine the acceptable level of correlation. Feature pairs with a correlation coefficient above this threshold (in absolute value) are considered highly correlated. For each pair of highly correlated features, one of the features is selected for removal.
vi. Model selection and training: Various machine learning models, K-nearest neighbors (KNN), AdaBoost, logistic regression, support vector machine (SVM), random forest, XGBoost, lightGBM, and Gaussian Naïve Bayes were implemented to classify liver disease status.
vii. **Cross-validation:** For each model, 5-fold cross-validation was applied to assess model performance robustly, ensuring that the model is not overfitting to the training data.
viii. **Hyperparameter tuning:** Hyperparameters for each model were optimized using techniques such as grid search and random search. Key hyperparameters for each model, such as the number of estimators for ensemble methods (random forest, AdaBoost, and XGBoost), regularization strength for logistic regression, kernel type for SVM, and learning rate for AdaBoost, were tuned to maximize the models' performance.

### 3.4 Handling the imbalanced dataset

Handling imbalanced datasets using different balancing techniques. Mainly, three methods are employed to handle imbalanced classes in a classification dataset.

- Undersampling.
- Oversampling.
- Hybrid sampling (a combination of both oversampling and undersampling).

The process of addressing class imbalance, specifically in the minority class, in machine learning is referred to as **Oversampling**. In this, we increase the number of samples in the minority class by introducing synthetic data. By doing so, it helps to obtain a more balanced representation of both the minority and the majority classes. Some oversampling methods involve duplicating existing samples, while others generate synthetic samples using various strategies. The use of oversampling techniques can enhance the performance of ML techniques.

The following oversampling techniques are used in this study:

➢ Upsampling: it is the process of randomly duplicating minority samples in the training dataset until the classes are balanced equally. This can be performed for all classes or just the classes that are underrepresented. The advantage of upsampling is that it does not exclude any data, which is important if your data is limited. It duplicates the already existing samples, which may lead to overfitting of the classification algorithms.

Pseudo code:
**Input**: The original train data
1. Choose an instance (xi) from the original train data.
2. Duplicate the instance at random in the minority classes.
3. Repeat this procedure till the required threshold is attained.
4. End
**Output**: Balanced version of the train data

➢ SMOTE: (Synthetic Minority Oversampling Technique) the primary use of this technique is for the balancing of data by synthesizing supplementary samples for the minority class. It randomly selects an instance from the minority class, identifies the nearest neighbors of the instance, and generates a synthetic instance by connecting them in the feature space. This technique involves the addition of new information to the data.

Pseudo code:
**Input:** M(samples of the minority class); N(synthetic sample amount); the number of k-nearest neighbors for i in range(N):
x = random.sample(M)//generating random sample
neighbors = k-nearest-neighbors(x)
y = random.sample(neighbors)
sample = x + (y - x) ^*^ random.uniform(0, 1) T.add(sample)
**Output:** T synthetic minority class samples

➢ ADASYN (Adaptive Synthetic Sampling) (29): this algorithm is based on SMOTE but with a specific focus on generating more examples for samples of the minority class, which have been deemed complex to learn. It seeks to alleviate class imbalance by generating synthetic samples based on the number of examples of the majority, for the minority class, in its nearest k-neighbors. This is achieved through linear interpolation between existing minority class samples.

The technique of balancing both classes by eliminating the majority class samples is known as **undersampling**. This technique mainly focuses on the majority class.

There is a possibility that we will lose data that is important to the dataset. As a result, this leads to a decrease in the performance of ML models. When the ratio of majority to minority classes is high, there is a chance that it will result in insufficient data for analysis. Additionally, as these techniques remove the majority class samples, the original randomness of the dataset is lost. Consequently, the resulting samples may not accurately represent the original target distribution.

The approach of combining oversampling with undersampling is known as hybrid sampling. Oversampling expands the data size through the addition of synthetic data, whereas undersampling reduces data points that cause loss. A hybrid approach combines both the oversampling technique and the undersampling technique, enhancing their strengths, as well as reducing their drawbacks.

➢ SMOTE-Tomek: Smote (Oversampler) + Tomek Links (Undersampler).

SMOTE's capacity for generating synthetic data for minority classes is combined with the ability of Tomek Links to remove majority class data that is closest to minority class data in this method. It can be used as an undersampling methodology to balance the dataset and remove the majority as well as the minority class samples.

TOMEK: given two samples that belong to different classes, Ei and Ej. The distance between Ei and Ej is d(Ei, Ej ). This tuple is a Tomek Link if there are no samples El, such that either d(Ei, El) or d(Ej, El) is less than d(Ei, Ej).

Pseudo code:
1. (Starting of SMOTE) From the minority class, choose random data.
2. The relative distance from the randomly selected data and its nearest k-neighbors is computed
3. Choose a random value in the range (0, 1) and multiply it by the estimated distance.
4. We obtain a simulated sample. Include this simulated sample in the minority class.
5. Steps 2 to 4 are repeated until the required proportion of the minority class is reached (conclusion of the SMOTE method).
6. (The start of Tomek links) Selection of the randomized majority class data.
7. Find the nearest neighbors of the randomized data. If it is minority class data, that is, Tomek Link is created, then remove the Tomek Link. (End)

➢ SMOTE-ENN (30): Smote (Oversampler) + ENN (Undersampler)

SMOTE increases the minority class samples at random by replicating them. The ENN stands for Edited Nearest Neighbor. First, it identifies the nearest k-neighbors of every data point and determines whether the majority class and observation class are the same. If they are not, both the k-nearest neighbor and observation are removed from the dataset. This method thus integrates both SMOTE for generating synthetic samples for the minority class, as well as ENN for eliminating majority class and minority class samples that are close.

Pseudo code:
1. (StartSMOTE) From the minority class, choose random data.
2. The relative distance from the randomly selected data and its nearest k-neighbors is computed.
3. Choose a random value in the range (0, 1) and multiply it by the estimated distance.
4. We obtain a simulated sample. Include this simulated sample in the minority class (end SMOTE).
5. (Beginning of ENN) The number of closest neighbors, k (*k* = 3 by default).
6. Find the nearest k-neighbor of the data point among all other data points in the observations.
7. The majority class of the nearest k-neighbors is returned.
8. If the majority class calculated and the observation class are not the same, both the k-nearest neighbor and the observation are removed from the dataset.
9. Steps 5–8 are repeated till the required proportion of all the classes is attained (End).

### 3.5 Model building and implementation

Train the model based on Classification Machine Learning Algorithms:

1. **Logistic regression:** the aim is the prediction of the probability of occurrence of a class label by analyzing the relationship between the independent and dependent variables sets. A sigmoid function is used for the estimation of the probability of a class label. The hyperparameters tuned included the regularization strength (C) and penalty (L1, L2).
2. **Support Vector Machine (SVM):** the aim of the SVM is the identification of a boundary or hyperplane that can differentiate classes. This boundary is chosen to maximize the margin, which represents the displacement between the boundary and its nearest observations. These observations, or data points, are known as support vectors, and they have an important role in defining the hyperplane. In a two-dimensional (2D) space, the hyperplane is essentially a line that divides the 2D plane into two sections, where both classes are located on opposing sides. We optimized the kernel type (linear, rbf), the regularization parameter (C), and the kernel coefficient (gamma) for the rbf kernel.
3. **Random forest:** also called random decision trees, is an ensemble learning technique. On each unique subset of the dataset, multiple decision trees are created, and through voting or averaging, their predictions are combined. Random forest uses multiple trees to increase the accuracy and avoid overfitting by considering predictions from each decision tree, resulting in a final output based on majority vote. The number of estimators (n_estimators) was tuned along with the maximum depth of trees (max_depth). “max_depth”: 5, “min_samples_split”: 2, “n_estimators”: 100
4. **KNN algorithm:** (k-nearest neighbors) it is a supervised, non-parametric ML technique utilizing proximity to classify/predict the cluster of individual data points. Data points that are near each other are considered similar, and this creates clusters based on these similar features. KNN also excels in capturing local relationships between data points, which is particularly useful in distinguishing between healthy and diseased liver conditions based on feature similarity. We tuned the number of neighbors (n_neighbors) along with the distance metric (euclidean, manhattan).
5. **AdaBoost:** (Adaptive Boosting) it is a boosting algorithm that integrates multiple weak models, creating a robust final model. AdaBoost is known for its ability to focus on misclassified instances, iteratively improving the classification performance by emphasizing difficult cases. The preferred estimator used with AdaBoost is decision trees, particularly decision stumps, which are decision trees with only one level (one split). In this technique, all data points are initially assigned equal weights, while higher weights are given to wrongly classified data points. These higher-weighted data points gain increased importance in training the subsequent models. The process continues iteratively until the model achieves a lower error. We optimized the number of estimators (n_estimators) and the learning rate (learning_rate).
6. **XGBoost**: (Extreme gradient boosting) it is a supervised ML technique, employing boosting for the creation of accurate models. It builds multiple weak models sequentially to achieve this. Initially, the model is trained, and then subsequent models attempt to correct the errors made by their predecessors. This process continues until the entire training dataset is correctly predicted or a maximum number of models is reached. We tuned the n_estimators, learning rate, and the maximum depth (max_depth) of trees.
7. **Decision tree algorithm:** it is a non-linear supervised ML technique used for classification as well as regression tasks. It divides the data into subdatasets recursively based on features and creates a tree-like structure. It selects the best feature for splitting the data at every tree-node. This process is continued until the result is further improved by splits or the pre-defined depth of the tree is reached. The decision boundaries for classification are represented by the final leaves of the tree. The hyperparameters included max_depth, min_samples_split, and the criterion (gini, entropy).
8. **Gaussian Naïve Bayes:** it is a classification methodology that uses a Gaussian distribution and a probabilistic approach. It is a specialized version of Naïve Bayes designed for scenarios where the features have continuous values, meaning they follow a Gaussian/normal distribution. Although this model has fewer hyperparameters, the variance smoothing parameter (var_smoothing) was optimized.
9. **Light GBM (Gradient boosting machine):** it is a gradient-boosting framework, which is available as open-source. It makes use of decision trees for the improvement of model efficiency and reduction of memory utilization. In contrast to XGBoost, where decision trees were built one level at a time, light GBM takes a leaf-wise approach. It also uses histogram binning of continuous features, which provides more speed-up than traditional gradient boosting. The hyperparameters optimized included the number of leaves (num_leaves), max_depth, and the learning rate.

Training the model on the ML algorithms discussed above, and then evaluating their performance using accuracy metrics, recall score, precision score, AUC-ROC, and F1 score (31). Because accuracy alone is not a good metric for imbalanced datasets.

➢ Confusion metrics: a useful tool for summarizing the results of an ML algorithm/model when tested on a test dataset. It is predominantly used for the evaluation of the efficacy of models that are supposed to predict category labels for input cases. During the testing phase, the matrix displays the counts of true positives (TP), false positives (FP), true negatives (TN), and false negatives (FN) generated by the model as represented in Table 2.
➢ Accuracy: the correctly classified observations or data points in a dataset, expressed as a percentage.

$$Accuracy=\frac{TP\;+\;TN}{TP\;+\;TN\;+\;FP\;+\;FN}$$

➢ Precision: (True positives/all predicted positives). It is a measure of a classifier's performance that tells how many positive predictions made by the model (classifier) are actually correct. It is a measure of the model's exactness. The lower precision values indicate that a high number of False Positives are there.

$$Precison=\frac{TP\;}{TP\;+FP}$$

➢ Recall: (True positives/-all actual positives). It is used to measure the proportion of actual positive labels. It is a measure of the completeness of a model. A low recall implies a large presence of false negatives.

$$Recall=\frac{TP\;}{TP\;+\;FN}$$

➢ F1 score: it serves as a weighted average of the precision and the recall. The F1-score is essential to restore the balance between the precision and recall.

$$F1\;Score\;=\frac{2*\;Recall\;\times \;Precision}{Recall\;+\;Precison}$$

➢ AUC_ROC: as the receiver operating characteristic (ROC) area under the curve (AUC) increases, the performance in terms of classification and differentiating between the positive class and the negative class improves.

**Table 2:** Confusion metrics.

		**Predicted value**	
		**Has liver disease**	**Does not have a liver disease**
**Actual value**	Have liver disease	True positive (TP)	False negative (FN)
	Does not have a liver disease	False positive (FP)	True negative (TN)

**Figure 5:**
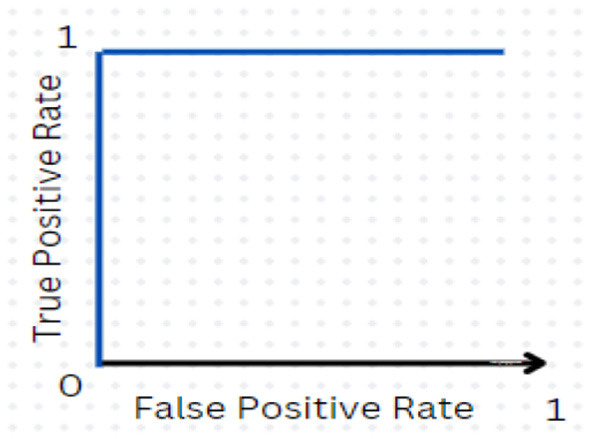
Ideal ROC curve.

The ROC–AUC is represented when AUC equals 1, which indicates the model correctly differentiates all the positive and negative class points. The ideal ROC curve is shown in Figure 5. A numerically greater value on the x-axis corresponds to a greater proportion of false positives as opposed to true negatives, while that on the y-axis corresponds to a greater proportion of true positives as opposed to false negatives. When the AUC becomes 0, all positives are predicted as negatives, and vice versa. For values of the AUC in the range (0.5, 1), the model is capable of separating positive class from negative class points. When AUC equals 0.5, the classifier fails at predicting classes for the data points, making random predictions.

## 4 Results and analysis

This section discusses the results we got from the implemented hybrid models, as well as a comprehensive comparative exploration of the performance of the models. It also shows the workflow of the system using the traditional approach and the results we get using the traditional approach (i.e., without handling an imbalanced dataset).

### 4.1 Using traditional ML models

Table 3 shows the result we got after implementing traditional ML algorithms such as SVM, Gaussian Naïve Bayes, logistic regression, AdaBoost, random forest, XGBoost, light GBM, KNN and decision tree on training and testing data.

**Table 3:** Accuracy of models on training and testing data.

**Model**	**Train accuracy (%)**	**Test accuracy (%)**
Logistic regression	72.36	71.05
SVM	70.39	73.68
Random forest	100	77.19
KNN	80.70	64.91
Decision tree	100	57.01
AdaBoost	81.79	71.92
XGBoost	100	72.80
Light GBM	100	69.2
Gaussian Naïve Bayes	57.89	51.75

**Figure 6:**
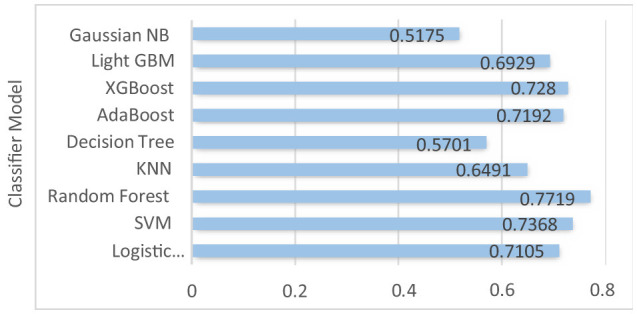
Accuracy of the implemented models.

Logistic regression, SVM, and AdaBoost show good performance, with accuracy of 71.05, 73.68, 77.19, 71.92, and 72.80% respectively. Random forest, decision tree, XGBoost, and light GBM models show 100% training accuracy. Overfitting occurs when an ML model fits too closely to a particular dataset. In simple terms, when a model is trained too much, the model starts memorizing the data instead of learning from it. Also, as the dataset is imbalanced, the implemented ML models are biased toward the majority class label. Using random forest for patients with no liver disease (minority class label), we have precision value: 55%; recall value: 37.93%; F1-score: 44.89%; and ROC–AUC score: 63.67%. We can see that the model is performing really poorly in predicting non-liver disease patient among liver patients. The graph of accuracy of implemented models is shown in Figure 6.

**Figure 7:**
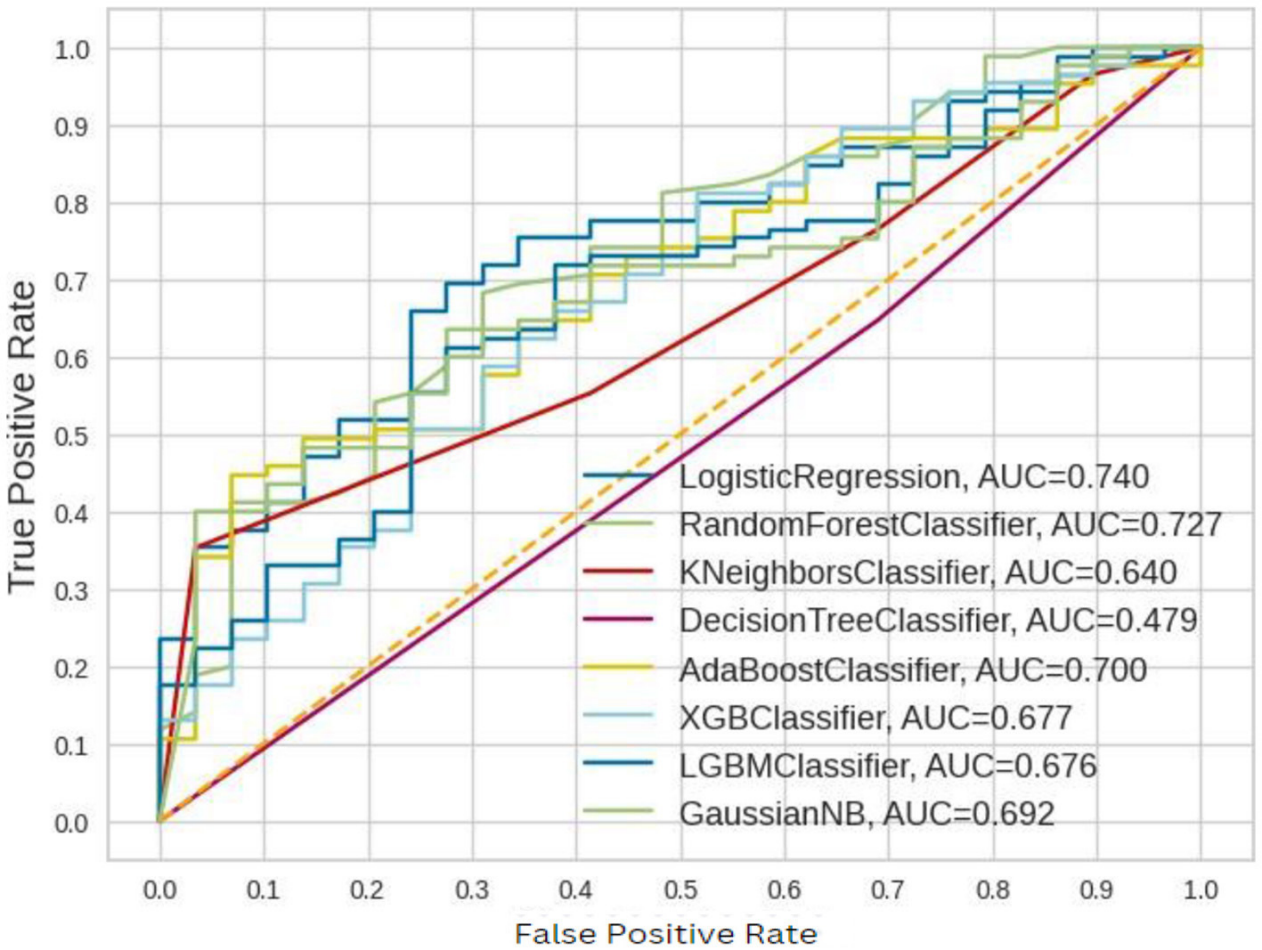
ROC–AUC for the implemented models before balancing.

The ROC–AUC for implemented models is depicted in Figure 7.

These ROC–AUC have a numerically greater value on the Y-axis, as discussed above, corresponds to a greater proportion of true positives as opposed to false negatives, as well as a greater proportion of false positives as opposed to true negatives. The implemented models are able to separate points from the positive and negative classes.

But these implemented models performed poorly.

### 4.2 Handling imbalanced dataset

- Using balancing techniques, such as SMOTE, SMOTE-Tomek, SMOTE-ENN, Upsampling, ADASYN, the data is balanced.
- The balanced data is divided in an 80:20 ratio with 80% train data and 20% test data.
- The models are trained on balanced data, and the implemented model is then tested on test data.
- The results we got for implemented classification techniques with different balancing techniques are shown in Table 4.

**Table 4:** Accuracy score of different balancing techniques on classifiers.

**Models**	**SMOTE (%)**	**SMOTE-ENN (%)**	**SMOTE-Tomek (%)**	**ADASYN (%)**	**Upsampling (%)**
Logistic regression	65.03	79.72	63.81	58.22	60.73
SVM	67.48	78.37	60.52	57.59	57.66
Random forest	**82.20**	**87.83**	**82.23**	75.94	**82.82**
KNN	73	**91.89**	74.34	72.78	68.71
Decision tree	74.84	**87.83**	75.65	66.45	**83.43**
AdaBoost	69.32	**91.89**	75	67.08	69.93
XGBoost	**77.91**	**90.54**	**82.89**	**73.41**	**82.20**
Light GBM	75.46	**90.54**	**82.89**	**75.31**	**82.82**
Gaussian Naïve Bayes	72.39	**90.54**	70.39	67.72	68.09

The bold values are indicating best results.

#### 4.2.1 Accuracy

**Figure 8:**
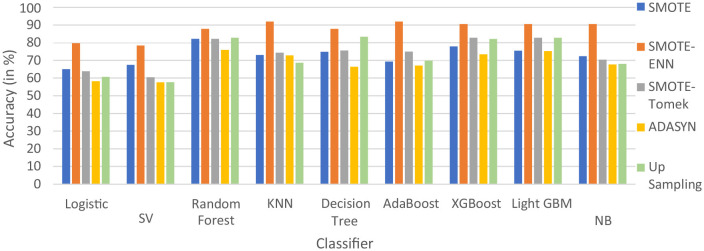
Accuracy of nine classifiers with different balancing techniques.

The hybrid models, KNN–SMOTE-ENN and AdaBoost-SMOTE-ENN show the highest accuracy of 91.89%. Using SMOTE techniques, the hybrid model, random forest-SMOTE, shows the highest accuracy of 82.2%. Using SMOTE-Tomek balancing techniques, the hybrid models, XGBoost-SMOTE-Tomek and light-GBM-SMOTE-Tomek, show the highest accuracy achieved is 82.89%. Using ADASYN balancing techniques, the hybrid model light-GBM-ADASYN shows the highest accuracy achieved is 75.31%. Using the upsampling technique, the hybrid model decision tree-upsampling shows the highest accuracy of 83.43%. Random forest, XgBoost, and light GBM show better accuracy in comparison to other ML algorithms with all the balancing techniques. The graph of the accuracy of all implemented hybrid models is shown in Figure 8 to analyze the results.

SMOTE-ENN performs well with all the implemented ML algorithms. Upsampling also shows good results, but it is necessary to check other evaluation metrics. This is because accuracy metrics are not a good measure of model performance for imbalanced datasets. In cases where the class distribution is imbalanced, relying solely on accuracy can lead to predictions that are biased, as it may favor the majority class. Instead, it is recommended to employ alternative metrics such as recall value, precision value, ROC—AUC, and F1 score to better assess the model's performance. The precision value, recall value, F1-score, and ROC–AUC score are calculated for the implemented hybrid model.

To statistically validate the improvements observed with hybrid models over traditional models, the Wilcoxon Signed-Rank Test was conducted. This non-parametric test evaluates the symmetry of the distribution of paired differences between accuracies. The Wilcoxon statistic was found to be 0.0, with a corresponding *p*-value of 0.0039, indicating a statistically significant improvement in hybrid models' performance (*p* < 0.05). The result supports the hypothesis that hybrid models provide robust enhancements in predictive accuracy for the ILPD dataset. For the precision value of the implemented hybrid models is displayed in Table 5.

**Table 5:** Precision value of different balancing techniques on classifiers.

**Precision values:**						
**Models**	**Label**	**SMOTE (%)**	**SMOTE-ENN (%)**	**SMOTE-Tomek (%)**	**ADASYN (%)**	**Upsampling (%)**
Logistic regression	0	64.91	76.78	60.78	58.51	56.14
	1	65.30	88.88	70	57.81	71.42
SVM	0	63.57	74.57	56.58	55.9	53.28
	1	91.30	93.33	82.60	62.85	80.76
Random forest	0	**80.39**	92.85	78.4	**76.19**	**76.04**
	1	**85.24**	81.25	87.5	**75.67**	**92.53**
KNN	0	69.74	**91.48**	70.65	70.1	65.16
	1	81.81	**92.57**	80	77.04	72.97
Decision tree	0	78.4	95	77.02	71.01	**75.75**
	1	70.66	79.41	74.35	62.92	**95.31**
AdaBoost	0	70.29	**95.34**	73.49	67.85	67.90
	1	67.74	**87.09**	76.81	66.21	71.95
XGBoost	0	78.35	93.18	**80.72**	73.25	**75.25**
	1	77.27	86.66	**85.50**	73.61	**92.42**
Light GBM	0	75.75	93.18	**80.72**	74.71	**76.59**
	1	75	86.66	**85.50**	76.05	**91.3**
Gaussian Naïve Bayes	0	68.25	89.58	64.54	63.02	61.01
	1	86.48	92.30	85.71	82.05	86.66

The bold values are indicating best results.

**Figure 9:**
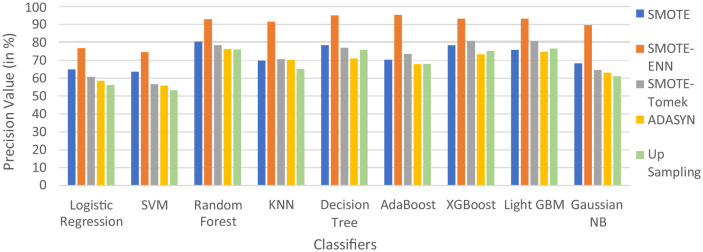
Precision score of nine classifiers with different balancing techniques for non-liver disease patient.

**Figure 10:**
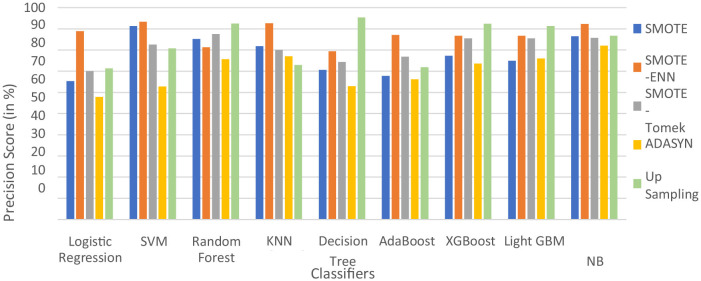
Precision score of nine classifiers with different balancing techniques for liver disease patients.

In Table 5, label “0” represents the patients not diagnosed with liver disease, and “1” represents patients with liver disease. The precision value for both labels is calculated and analyzed. The hybrid model KNN- SMOTE-ENN shows the highest precision score of 91.48% for patients not diagnosed with liver disease and 92.57% for patients with liver disease. KNN excels in capturing local relationships between data points, which is particularly useful in distinguishing between healthy and diseased liver conditions based on feature similarity. The hybrid models designed using upsampling techniques show lower precision values for patients without liver disease as opposed to those with liver disease, as a result of overfitting. In other balancing techniques, the difference in precision values is not that much. Using the SMOTE technique, the hybrid model random forest-SMOTE shows the highest precision score of 80.39% for patients not diagnosed with liver disease and 85% for patients with liver disease. Using the SMOTE-Tomek technique, the hybrid models, XGBoost-SMOTE-Tomek and light GBM-SMOTE-Tomek, show the highest precision value of 80.72% for patients not diagnosed with liver disease and 85.5% for patients diagnosed with liver disease. Using the ADASYN technique, the hybrid model random forest-ADASYN shows the highest precision value of 76.19% for patients not diagnosed with liver disease and 75.67% for patients diagnosed with liver disease, among all implemented models. Using upsampling technique, the hybrid model decision tree gives the highest precision value of 75.75% for patients not diagnosed with liver disease and 95.31% for patients diagnosed with liver disease. The individual graph of precision value for patients not diagnosed with liver disease and patients diagnosed with liver disease is shown in Figures 9, 10.

From the graph, it can be seen that up-sampling techniques show good precision value for patients diagnosed with liver disease and perform poorly for patients not diagnosed with liver disease. The SMOTE-ENN balancing techniques show good precision score for both patients who are diagnosed with liver disease and those who are not. Table 6 shows the recall value of implemented hybrid models.

**Table 6:** Recall value of different balancing techniques on classifiers.

**Recall values**	
**Models**	**Label**	**SMOTE (%)**	**SMOTE-ENN (%)**	**SMOTE-Tomek (%)**	**ADASYN (%)**	**Upsampling (%)**
logistic Regression	0	81.31	95.55	80.51	67.07	82.05
	1	44.44	55.17	46.66	48.68	41.1
SVM	0	97.80	97.77	94.8	84.14	93.58
	1	29.16	49.27	25.33	28.94	24.70
Random forest	0	**90.10**	86.67	**89.61**	**78.04**	**93.58**
	1	**72.22**	89.65	**74.66**	**73.68**	**72.94**
KNN	0	91.2	**95.55**	84.41	82.92	74.35
	1	50	**86.20**	64	61.84	63.52
Decision tree	0	75.82	84.44	74.02	59.75	**96.15**
	1	73.61	93.10	77.33	73.68	**71.76**
AdaBoost	0	78.02	**91.11**	79.22	69.51	70.51
	1	58.33	**93.10**	70.66	64.47	69.41
XGBoost	0	83.51	91.11	**87.01**	76.82	**93.58**
	1	70.83	89.65	**78.66**	69.73	**71.76**
Light GBM	0	82.41	91.11	**87.01**	79.26	**92.30**
	1	66.66	89.65	**78.66**	71.05	**74.11**
Gaussian Naïve Bayes	0	94.5	95.55	92.2	91.46	92.34
	1	44.44	82.75	48	42.10	45.88

The bold values are indicating best results.

**Figure 11:**
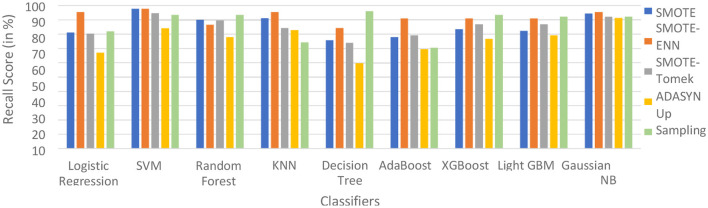
Recall score of nine classifiers with different balancing techniques for non-liver disease patients.

**Figure 12:**
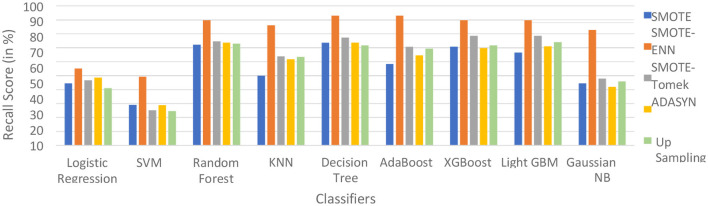
Recall Score of 9 classifiers with different balancing techniques for liver disease patients.

The hybrid model AdaBoost-SMOTE-ENN shows the highest recall score of 91.11% for patients not diagnosed with liver disease and 93.1% for patients diagnosed with liver disease, among all implemented models. The other hybrid models which have good recall value for both patients who are not diagnosed for liver disease as well as those that are KNN–SMOTE-ENN, XGBoost-SMOTE-ENN, and light GBM-SMOTE-ENN. These hybrid models have a recall value of more than 90% for non-liver disease patients and a recall value of more than 86% for liver disease patients. The random forest-SMOTE-ENN hybrid models show a good recall value of more than 85% for both patients who are not diagnosed with liver disease as well as those who are diagnosed with liver disease. The individual graphs of recall value for both patients who are not diagnosed with liver disease are shown in Figures 11, 12.

SMOTE-ENN shows better performance for patients not diagnosed with liver disease and for patients who are diagnosed with liver disease. The hybrid models that use other balancing techniques show poor recall for liver disease patients. The F-1-score is listed in Table 7.

**Table 7:** F-1-score different balancing techniques on classifiers.

**F-1-score**	
**Models**	**Label**	**SMOTE (%)**	**SMOTE-ENN (%)**	**SMOTE-Tomek (%)**	**ADASYN (%)**	**Upsampling (%)**
Logistic regression	0	72.19	85.14	69.27	62.5	66.66
	1	52.89	68.08	56	52.85	52.23
SVM	0	77.05	84.61	70.87	67.94	67.90
	1	44.21	63.63	38.77	39.63	37.83
Random forest	0	**84.97**	89.65	83.63	**77.1**	**83.90**
	1	**78.19**	85.24	80.57	**74.66**	**81.57**
KNN	0	79.04	**93.47**	76.92	75.97	69.46
	1	62.06	**89.28**	71.11	68.61	67.92
Decision tree	0	77.09	89.41	75.49	64.9	**84.74**
	1	72.1	85.71	75.81	67.87	**81.87**
AdaBoost	0	73.95	**93.18**	76.25	68.67	69.18
	1	62.68	**90**	73.61	65.33	70.65
XGBoost	0	80.85	92.13	**83.75**	75.90	83.42
	1	73.91	88.13	**81.94**	71.62	80.79
Light GBM	0	78.94	92.13	**83.75**	**76.92**	83.72
	1	70.58	88.13	**81.94**	**73.46**	81.81
Gaussian Naïve Bayes	0	79.26	92.47	75.93	74.62	73.46
	1	58.71	87.27	61.15	55.65	60

The bold values are indicating best results.

The hybrid model KNN–SMOTE-ENN shows the highest F1-score of 93.47% for patients not diagnosed with liver disease and 89.28% for patients diagnosed with liver disease, among all implemented models. The AdaBoost-SMOTE-ENN hybrid models also show a good F1-score of 93.18% for patients not diagnosed with liver disease and 90% for patients diagnosed with liver disease, among all implemented models.

The combination of SMOTE-ENN with KNN and AdaBoost was strategically chosen for the ILPD dataset to address issues related to class imbalance and noisy data, which are common in medical datasets like liver disease detection. The rationale for using this combination is as follows:

The ILPD dataset exhibits an imbalance between the number of healthy individuals and those with liver disease. SMOTE is used to synthetically generate samples for the minority class (patients with liver disease), thus helping to balance the dataset. This ensures that the models do not become biased toward the majority class (healthy individuals) and can better identify the minority class, which is critical in medical diagnosis tasks where detecting the diseased class is of utmost importance. Additionally, Edited Nearest Neighbors (ENN) is applied to remove noisy or misclassified instances from the dataset. By cleaning the data, ENN improves the quality of the training data, making the model more robust and reducing the likelihood of overfitting, which is especially important in medical datasets that may contain ambiguous or erroneous entries. KNN benefits from the balanced and cleaned dataset provided by SMOTE-ENN, as it relies on proximity between instances to make predictions. In the case of the ILPD dataset, having a well-balanced representation of both healthy and diseased classes allows KNN to more accurately classify the liver disease cases, as the model can better identify the true similarities between instances. Additionally, the cleaning performed by ENN helps KNN avoid the influence of noisy or irrelevant data points, improving its overall accuracy. AdaBoost is an ensemble learning method that combines several weak classifiers to create a strong classifier, with a focus on misclassified instances by increasing their weight in subsequent iterations. When combined with SMOTE (for balancing) and ENN (for cleaning), AdaBoost is better able to focus on the harder-to-classify minority class, improving its performance on liver disease detection. By addressing both class imbalance and noise, the ensemble method is more likely to correctly classify patients with liver disease, which is crucial for medical decision-making. The hybrid model KNN–SMOTE-ENN shows the highest F1-score of 93.47% for patients not diagnosed with liver disease and 89.28% for patients diagnosed with liver disease, among all implemented models.

The hybrid models created using SMOTE, SMOTE-Tomek, and upsampling balancing techniques show almost similar F1-score for patients who are and are not diagnosed with liver disease. Using the SMOTE technique, the random forest-SMOTE hybrid model shows the highest F1-score of 84.97% for patients not diagnosed with liver disease and 78.19% for patients diagnosed with liver disease.

**Figure 13:**
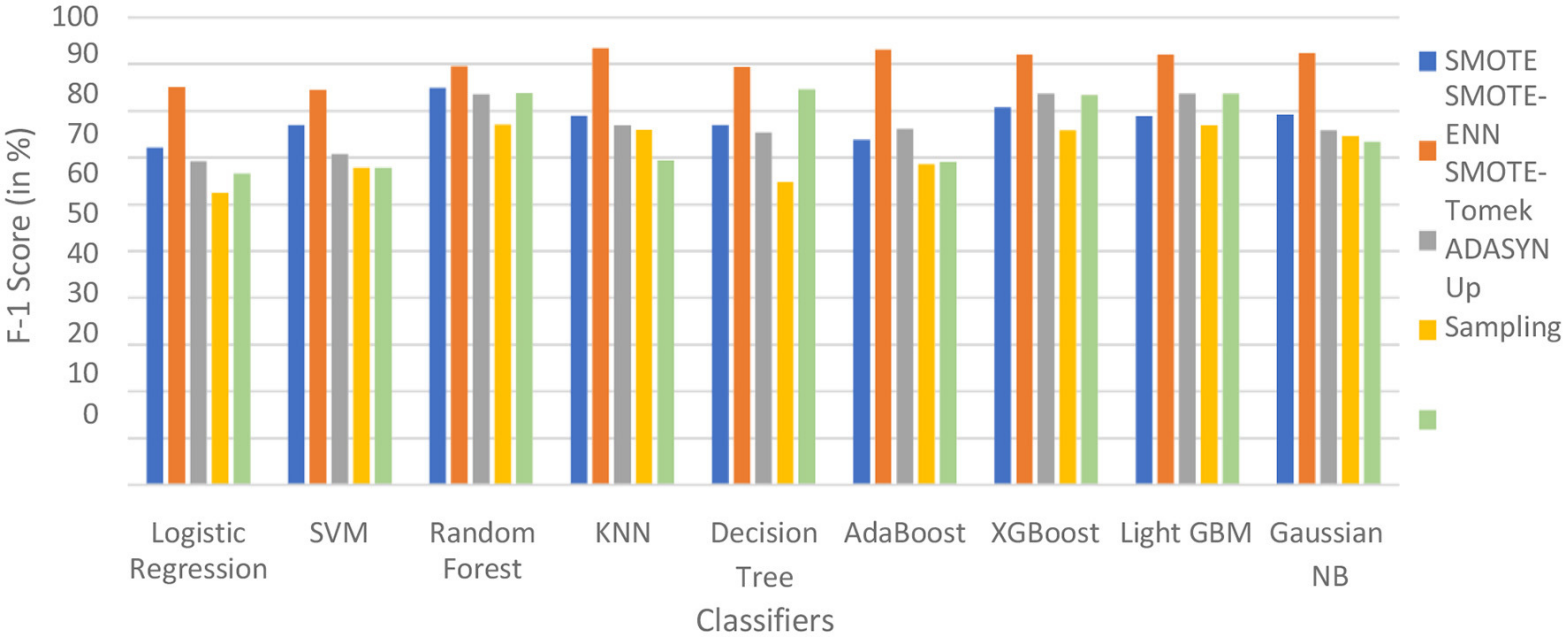
F1-score of nine classifiers with different balancing techniques for non-liver disease patients.

Using the SMOTE-Tomek technique, the hybrid models XGBoost-SMOTE-Tomek and light GBM-SMOTE-Tomek show the highest F1-score of 83.75% for patients not diagnosed with liver disease and 81.94% for patients diagnosed with liver disease. Using the up-sampling technique, the hybrid model Decision-Tree-Up-sampling gives the highest accuracy of 84.74% for patients not diagnosed with liver disease and 81.87% for patients diagnosed with liver disease. The individual graphs of F1-score for patients with and without liver disease are shown in Figure 13.

**Figure 14:**
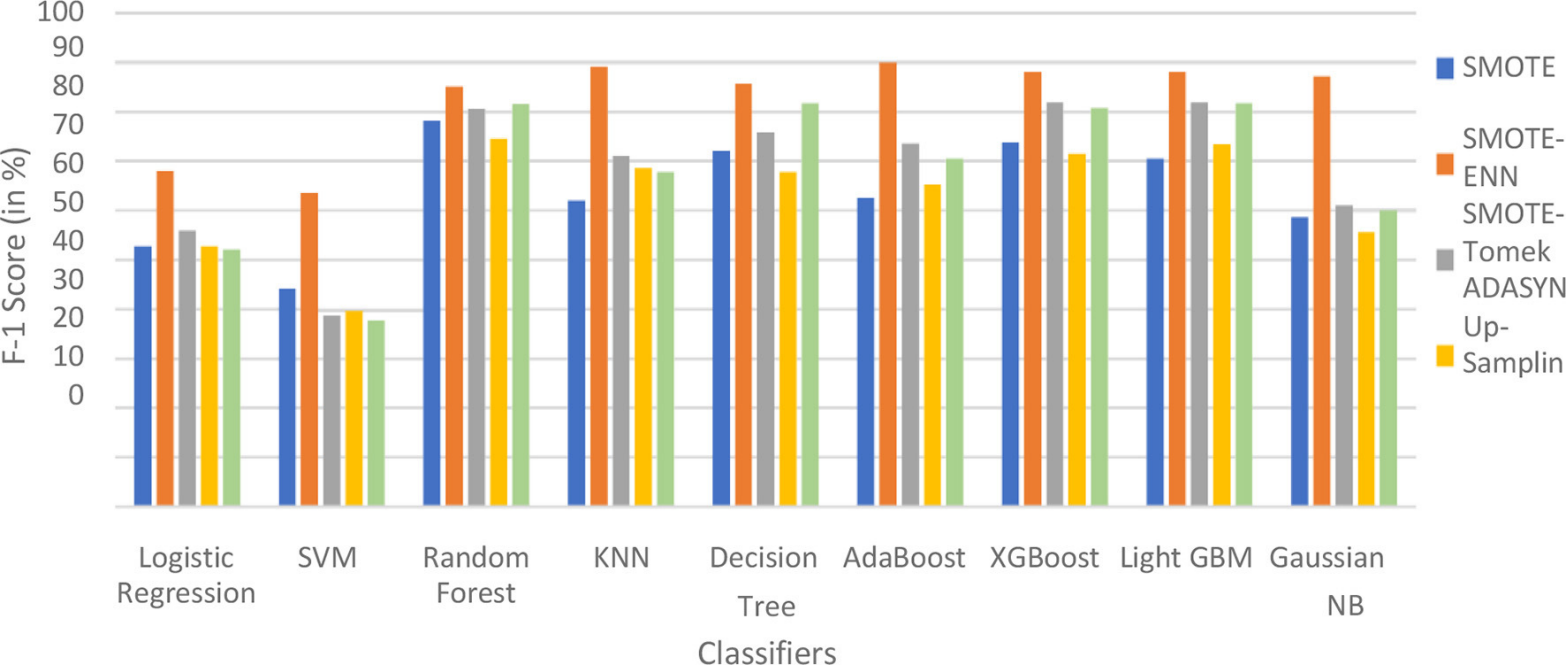
F1-score of nine classifiers with different balancing techniques for liver disease patients.

The hybrid models that use the SMOTE-ENN technique show a better F1-score than other hybrid models. The ROC scores of the implemented hybrid models are provided in Figure 14 and Table 8.

**Table 8:** ROC–AUC Score of different balancing techniques on classifiers.

**Models**	**SMOTE (%)**	**SMOTE-ENN (%)**	**SMOTE-Tomek (%)**	**ADASYN (%)**	**Upsampling (%)**
Logistic regression	62.88	75.36	63.59	57.87	61.61
SVM	63.48	73.02	60.06	56.54	59.14
Random forest	**81.16**	88.16	**82.13**	**75.86**	83.26
KNN	70.60	**90.88**	74.20	72.38	68.94
Decision tree	74.71	88.77	75.67	66.72	**83.95**
AdaBoost	68.17	**92.10**	74.94	66.99	69.96
XGBoost	77.17	90.38	**82.83**	73.28	82.67
Light GBM	74.54	90.38	**82.83**	75.16	83.21
Gaussian Naïve Bayes	69.47	89.15	70.1	66.78	69.09

The bold values are indicating best results.

#### 4.2.2 ROC–AUC Score

**Figure 15:**
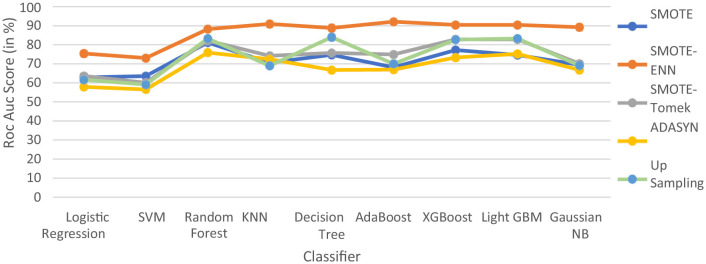
ROC–AUC score of nine classifiers with different balancing methodologies.

The hybrid model AdaBoost-SMOTE-ENN shows the highest ROC–AUC score of 92.1% among all implemented models. Using the SMOTE technique, the random forest-SMOTE hybrid model shows the maximum ROC–AUC score of 81.16%. Using the SMOTE-Tomek technique, the random forest-SMOTE, XGBoost-SMOTE-Tomek, and light-GBM-SMOTE-Tomek hybrid models show the highest ROC–AUC score of 82.83%. Using the ADASYN technique, the random forest-ADASYN hybrid model shows the maximum ROC–AUC score of 75.86%. Using the SMOTE technique, the decision-upsampling hybrid model shows the highest ROC–AUC score of 83.95%. The graph of the ROC–AUC score for the implemented models is shown in Figure 15.

The hybrid model, which uses the SMOTE-ENN technique, shows better results than other implemented models. SMOTE-ENN is a two-step technique that integrates SMOTE with the Edited Nearest Neighbors (ENN) algorithm in order to enhance classifier performance on imbalanced data. In this approach, Synthetic Minority Oversampling Technique (SMOTE) creates synthetic examples for the minority class by interpolating between existing instances and their nearest neighbors. This method expands the minority class dataset without merely duplicating samples, addressing the problem of limited training data while reducing the risk of overfitting, often associated with simple sample replication. Following this, ENN (Edited Nearest Neighbors) enhances the dataset by eliminating noisy and ambiguous instances from both majority and minority classes, particularly those misclassified by their k-nearest neighbors. This step not only removes inaccurately generated synthetic points but also filters out outliers and borderline cases in the original dataset that might blur decision boundaries. By combining these techniques sequentially, the process achieves a more balanced class distribution and cleaner data, ultimately improving classification accuracy compared to using SMOTE or ENN individually.

**Figure 16:**
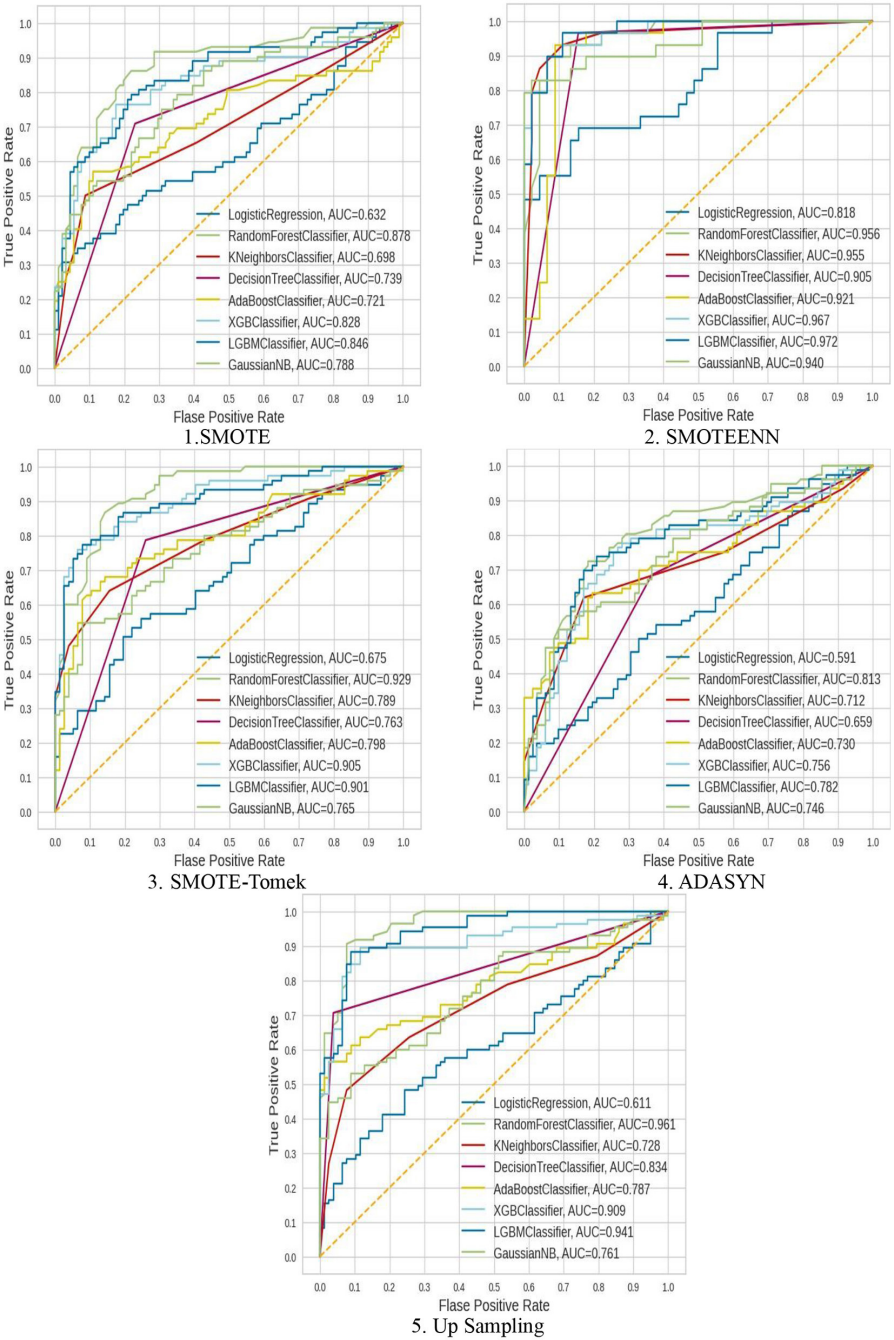
ROC–AUC of all the balancing techniques used.

The dataset has a limited number of minority class instances and contains noisy and overlapping data points, particularly due to physiological and biochemical variations among individuals. Hence, the hybrid approach of SMOTE-ENN is best suited as it balances the dataset while simultaneously improving the quality of samples. The hybrid models, which use SOMTE-Tomek and upsampling, show similar ROC–AUC score. Among the implemented balancing techniques, ADASYN shows the lowest performance. Classification algorithms are assessed using multiple metrics, including accuracy, recall, precision, ROC–AUC, and F1 score. Now, ROC–AUC of all the balancing techniques on implemented models are shown in Figure 16.

The ROC–AUC of SMOTE-ENN shows that SMOTE-ENN is most sensitive, that is, it can easily distinguish between class labels.

From the result table, graphs, and ROC–AU, we can conclude the following:

- Upsampling also underperformed with different classification techniques because it creates duplicate copies of existing data, resulting in overfitting in the classification.
- SMOTE-Tomek and SMOTE show similar performance. In some cases, both underperform. SMOTE underperformed in comparison to SMOTE-ENN because it lacks flexibility and did over generalization. SMOTE-Tomek uses SMOTE and Tomek to oversample the minority class and remove the majority class samples that are close to minority class samples, respectively.

Among all the balancing techniques, SMOTE-ENN gives a better accuracy score, precision value, recall value, F1-score, and ROC–AUC score. The hybrid models KNN–SMOTE-ENN, AdaBoost-SMOTE-ENN are suggested. The hybrid model KNN–SMOTE-ENN gives accuracy: 91.89%, precision value: 91.48%, recall value: 95.55%, F1-score: 93.47%, and ROC–AUC score: 90.88%. The AdaBoost-SMOTE-ENN hybrid model shows accuracy: 91.89%, precision value: 95.34%, recall value: 91.11%, F1-score: 93.18%, and ROC–AUC Score: 92.10%.

Some other models that performed well are XGBoost, light GBM, and Gaussian Naïve Bayes, with an accuracy of almost 90%. The random forest-SMOTE-ENN hybrid model shows Accuracy score, recall value, precision value, F1-score and ROC–AUC score of 87.82, 92.85, 89.01, 89.65, and 88.16%, respectively. The XGBoost-SMOTE-ENN hybrid model shows accuracy score, precision value, recall value, F1-score, and ROC–AUC score of 90.54, 93.18, 91.11, 92.13, and 90.38%, respectively. The light GBM-SMOTE-ENN hybrid model shows accuracy, precision, recall, F1-score, and ROC–AUC of 90.54, 93.18, 91.11, 92.13, and 90.38%, respectively. The Gaussian-NB-SMOTE-ENN hybrid model shows accuracy, precision, recall, F1-score, and ROC–AUC of 90.54, 89.58, 95.55, 92.47, and 92.1%, respectively.

**Figure 17:**
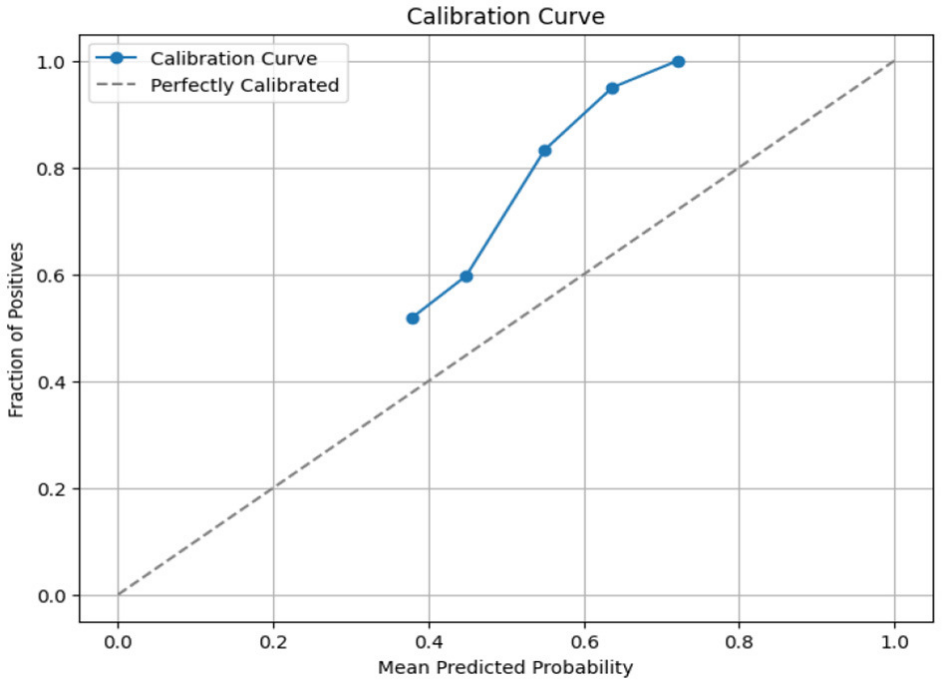
Calibration curve of Adaboost with SMOTE-ENN.

Since KNN, AdaBoost, XGBoost, light GBM, and Gaussian Naïve Bayes gave high accuracy scores with the SMOTE-ENN data balancing techniques. Hence, we have also tried to find the Brier score loss and the calibration curves for this combination to analyze the results deeply. Brier score is a type of **evaluation metric for classification tasks where lower values indicate better predictive reliability**.


(1) $$\begin{array}{l} BS=\;\frac{1}{N}\;\overset{N}{\underset{t=1}{\sum }}{\left(f_{t}-o_{t}\right)}^{2} \end{array}$$

where f_t_ is the predicted value and o_t_ is the observed value; N is the number of observations. The Brier score calculates the mean squared error between predicted probabilities and the observed values (actuals). **AdaBoost gave a Brier score loss of 0.2256, a**mong the evaluated models as shown in Table 9. AdaBoost achieved the lowest Brier score loss, indicating superior probability estimation and model calibration. Ensemble-based models (AdaBoost, XGBoost, and light GBM) performed better in terms of probability calibration compared to non-ensemble models (KNN, Naïve Bayes). Also, we have obtained the calibration curve for the AdaBoost with SMOTE-ENN. The calibration curve is a graphical representation of how well the predicted probabilities from the AdaBoost model align with actual observed outcomes, as shown in Figure 17. From the calibration curve, we can observe that for lower predicted probabilities, which are below 0.4, the fraction of positives is lower than expected, indicating under-confidence in predictions. However, for mid-to-high probability predictions (0.4–0.8), the model overestimates the likelihood of positive cases, and for very high probability values (above 0.8), the model is relatively well-calibrated.

**Table 9:** Brier score loss and calibration curves.

**Models**	**Balancing techniques**	**Brier score loss**
KNN	**SMOTE-ENN**	0.3435
AdaBoost	**SMOTE-ENN**	0.2256
XGBoost	**SMOTE-ENN**	0.2602
Light GBM	**SMOTE-ENN**	0.2935
Gaussian Naïve Bayes	**SMOTE-ENN**	0.3856

We also applied Recursive Feature Elimination for feature selection on Adaboost with SMOTE-ENN, which performs by recursively removing the least important features and re-fitting the model until the desired number of features is reached. It helps in selecting the most relevant features, improving model interpretability, and also prevents overfitting by removing redundant or noisy features. The Brier score loss for Adaboost with SMOTE-ENN became 0.2168. The RFE training time comes out to be 0.0310 s, and the AdaBoost training time is 0.2032 s.

**Figure 18:**
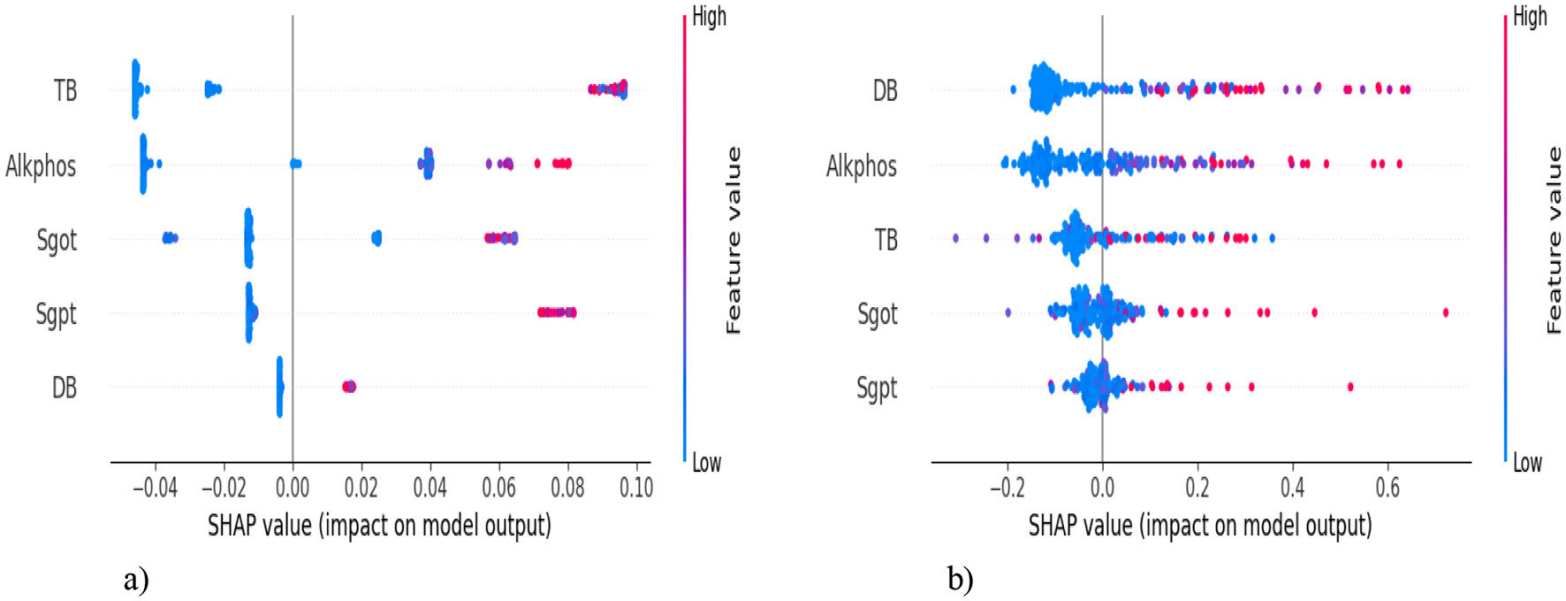
SHAP summary plot. **(a)** SMOTE-ENN with Adaboost. **(b)** SMOTE-ENN with KNN.

We analyze our best model's predictions using explainable artificial intelligence (XAI) techniques, such as SHapley Additive exPlanations (SHAP), which are used to explain machine learning model predictions in a mathematically sound and interpretable way, and help to gain transparency into its decision-making process. SHAP helps determine **which features contribute the most** to a model's predictions. SHAP provides both **global** (overall importance) and **local** (individual predictions) explanations. Figure 18 shows the **SHAP summary plot (feature importance)**, which shows how much each feature **impacts** predictions on average and also helps in feature selection. In Figure 18a, the top-5 features are listed on the y-axis [TB, Alkphos, SGOT, SGPT, Direct Bilirubin (DB)], and the SHAP value is represented on the x-axis. This indicates the average impact of each feature on the model's prediction. Positive values mean the feature contributes positively to the prediction, while negative values contribute negatively. The **feature value** is shown by the color of each dot. Red indicates high values, while blue indicates low values of the feature. TB has a significant positive impact on the model output. Most data points cluster on the right side of the zero line, indicating positive SHAP values. The color gradient suggests that high TB values (red dots) correspond to higher positive SHAP values. It indicates that the samples with high TB levels largely increase the predicted outcome (likely indicating liver disease). Alkphos shows a more varied impact. The data points appear on both sides of the zero line, with a notable concentration on the positive side. Higher Alkphos values are linked to positive SHAP values, while lower values have a slightly negative to negligible impact. Samples with higher Alkphos values tend to increase the predicted outcome, but not as significantly as TB. DB has a smaller impact compared to the other features, with data points more tightly clustered around the zero line. The gradient indicates that higher DB values might have a slightly positive influence, though the overall impact is less pronounced. In Figure 18b, DB shows a wide distribution of SHAP values but mainly clustered around SHAP value zero. Most of the points are clustered around zero, with some having a positive impact and some having a negative impact.

#### 4.2.3. Proposed hybrid ensemble model

We have designed a hybrid Machine learning model to predict liver disease using the Indian Liver Patient Dataset (ILPD). The hybrid model involves the combination of Recursive Feature Elimination (RFE) for feature selection, SMOTE-ENN to address the problem of data imbalance, and ensemble learning.

The ILPD dataset is first preprocessed. The missing values are filled with the mean of their respective columns following the imputation technique, and the target variable is transformed into a binary format to prepare it for the binary classification. The dataset is split into the train and test using the train_test_split with an 80:20 ratio. Recursive Feature Elimination (RFE) is applied to select the most important and relevant set of features. This technique recursively removes features and builds a model on the remaining attributes. It uses a RandomForestClassifier as the base estimator to determine feature importance. n_features_to_select=7 specifies that the RFE should select the top-7 features, namely [“age,” “total_bilirubin,” “alkaline_phosphatase,” “alamine_aminotransferase,” “aspartate_aminotransferase,” “total_proteins,” “albumin”]. The ILPD dataset is a highly imbalanced dataset; hence, SMOTE-ENN is applied for data balancing *after* feature selection. Then, the scaling of the feature is performed using MinMaxScaler to a range between 0 and 1. The training and testing datasets are re-split after resampling and scaling.

Ensemble learning is performed using a VotingClassifier, which integrates predictions from multiple classifiers to enhance predictive accuracy. Defines two base classifiers: RandomForestClassifier and GradientBoostingClassifier. Both are initialized with n_estimators=100 (meaning 100 trees/estimators in each ensemble) and a random_state for reproducibility. The methodology involves defining two base classifiers: RandomForestClassifier and GradientBoostingClassifier. These classifiers are chosen for their ability to capture complex patterns in the data. The VotingClassifier employs soft voting, which means it predicts the class label based on the predicted probabilities from each classifier rather than simply taking a majority vote. After training the VotingClassifier on the training dataset, predictions are made on the test set. The ensemble model's performance is evaluated using various metrics, including accuracy, ROC–AUC score, and Brier score loss. These metrics provide insights into how well the ensemble model generalizes to unseen data.

**Figure 19:**
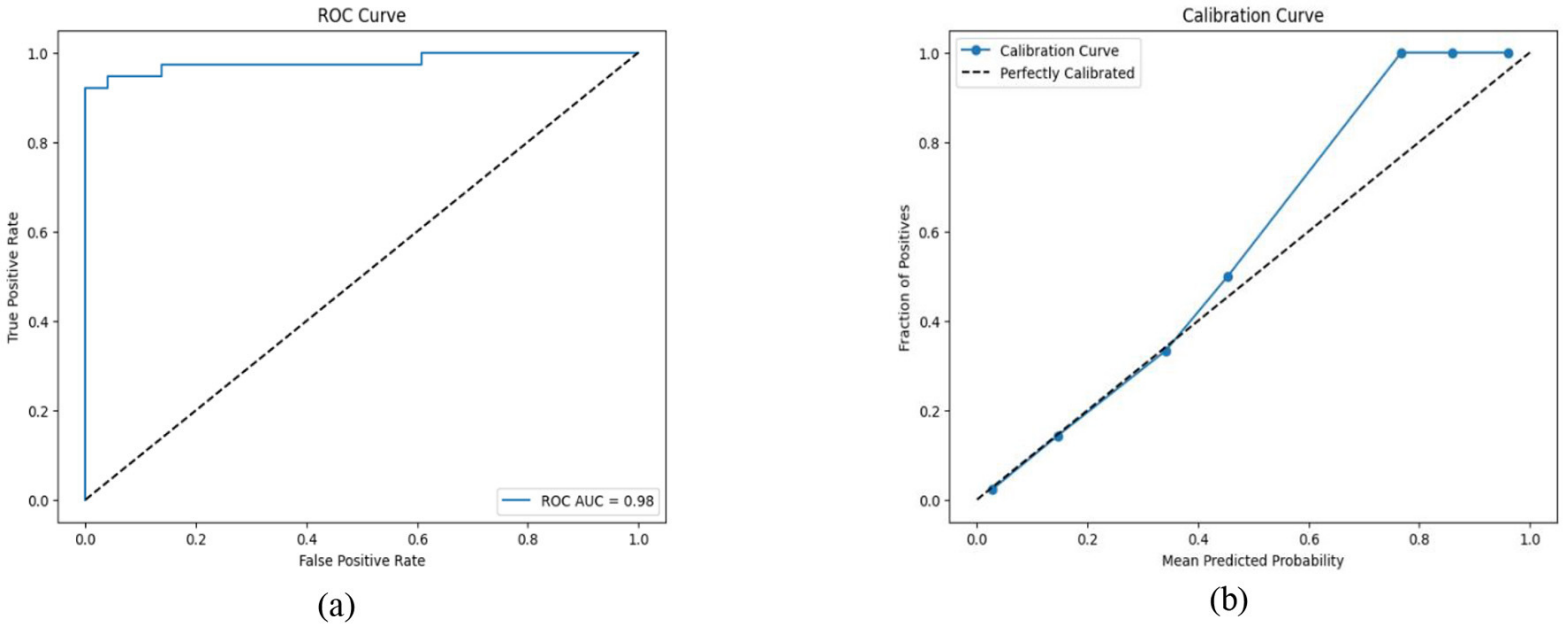
**(a)** ROC–AUC; **(b)** Calibration curve of the proposed hybrid ensemble model.

Table 10 shows the performance of the proposed hybrid ensemble model on the ILPD and BUPA Liver Disorder Dataset. For the ILPD dataset, the model achieves an overall accuracy of 93.2%, whereas for the BUPA dataset, the model attains an accuracy of 95.4%. The Brier score loss for the ILPD dataset is 0.032 and 0.031 for the BUPA Liver Disorder Dataset. The hybrid ensemble model demonstrates strong performance on both datasets, with high accuracy, precision, recall, and F1-scores, coupled with low Brier score loss and excellent ROC–AUC scores. The Figure 19 shows the ROC–AUC b) Calibration Curve. Figure 19a shows a receiver operating characteristic (ROC) curve with a very high area under the curve (AUC) score of 0.98. This signifies that the model achieves a high true positive rate (TPR) while maintaining a low false positive rate (FPR) across a wide range of thresholds. This indicates that the classification model performs exceptionally well at distinguishing between the two classes (positive and negative). In Figure 19b, the calibration curve shows that the blue line closely follows the dashed diagonal line for mean predicted probabilities in the range of ~0–0.4. This indicates that the model is well-calibrated for predictions in this range; when the model predicts a low probability, it is indeed a low probability event.

**Table 10:** The performance of the hybrid ensemble model for the ilpd and bupa liver disorder dataset.

**Dataset**	**Label**	**Accuracy (%)**	**Precision**	**Recall**	**F1-score**	**Brier score loss**	**ROC–AUC**
ILPD	0	93.2	0.94	0.97	0.95	0.032	0.99
	1		0.92	0.93	0.93		
BUPA	0	95.4	0.98	0.96	0.97	0.031	0.99
	1		0.95	0.97	0.94		

The proposed hybrid model is compared with the recent existing studies carried out for liver disease prediction on the ILPD datasets. We have considered the accuracy as the key criterion to perform the comparison among all the studies and presented them in Table 11. The proposed hybrid model (SMOTE-ENN + AdaBoost) demonstrates significant improvement over previous studies on the ILPD dataset, achieving the highest accuracy (91.89%) among all approaches compared in the table. This improvement can be attributed to the effective handling of class imbalance using SMOTE-ENN. The powerful ensemble learning capabilities of AdaBoost optimize classification performance. The results clearly establish the superiority of the proposed approach for liver disease diagnosis using ILPD data, making it a robust solution for clinical applications and research purposes in this domain.

**Table 11:** Comparison of our proposed hybrid models with the other previous studies.

**Authors**	**Dataset**	**Classifiers**	**Accuracy**
Singh et al. (9)	IPLD	Logistic regression (LR), SVM, and KNN	LR: 73.97% KNN: 73.97% SSVM:71.79%
Keerthana et al. (10)	ILPD	LR	LR: 85.96%
Fernando et al. (14)	ILPD	Random forest (RF), multilayer perceptron (MLP), KNN, and SVM	RF: 69% KNN: 67% SVM: 74% MLP: 68%
Gupta et al. (23)	ILPD	Logistic regression (LR), decision tree (DT), KNN, RF, gradient boosting (GB), XGBoost, light GBM	LR: 57%, NB: 54% DT: 61, RF: 63, XGBoost: 60%, AdaBoost: 62% LGBM: 63% KNN: 57%
Amin et al. (33)	ILPD	Logistic KNN Random forest SVM MLP Ensemble	55.4 67.9 88.1 67.9 83.53 82.09
Altaf et al. (34)	ILPD	Voting ensemble	73.56
**Proposed**	ILPD	Hybrid (SMOTE-ENN AdaBoost) Hybrid ensemble Model	91.89% 93.2%

## 5 Conclusion

The early and precise prediction of liver disease has been examined and analyzed. The dataset we chose is an imbalanced dataset. We used balancing methodologies, such as upsampling, ADASYN, SMOTE, SMOTE-ENN, and SMOTE-Tomek, for balancing the dataset. After that, machine learning models are implemented with all the different balancing techniques mentioned.

After performing a thorough comparative analysis, we observed that SMOTE-ENN yields better results compared to other balancing techniques. The hybrid models KNN–SMOTE-ENN and AdaBoost-SMOTE-ENN are suggested for the prediction of liver disease.

- KNN with SMOTE-ENN gives accuracy: 91.89%, precision score (patients not diagnosed with liver disease): 91.48%, precision value (patients diagnosed with liver disease): 92.57%, recall value (patients not diagnosed with liver disease): 95.55%, recall value (patients diagnosed with liver disease): 86.20%, F1-score (patients not diagnosed with liver disease): 93.47%, F1-score (patients not diagnosed with liver disease): 89.28%, and ROC–AUC score: 90.88%.
- AdaBoost with SMOTE-ENN gives accuracy: 91.89%, precision value (patients not diagnosed with liver disease): 95.34%, precision (patients diagnosed with liver disease): 87.09%, recall value (patients not diagnosed with liver disease): 91.11%, recall value (patients diagnosed with liver disease: 93.10%, F1-score (patients not diagnosed with liver disease): 93.18%, F1-score (patients diagnosed with liver disease): 90%, and RUC–AUC score: 92.10%.

From the results, it can be concluded that the probability of having liver disease can be predicted with an accuracy of more than 90%. The results we obtained show that some of the techniques, except the KNN model, may perform well under specific parameters, combined with the AdaBoost model and the SMOTE-ENN balancing technique, accurately predicted patients with liver disease.

However, the inference time for KNN–SMOTE-ENN is relatively high due to the need for real-time distance calculations, which may not be ideal for real-time applications. This is because the training time for KNN–SMOTE-ENN is influenced by the KNN algorithm's reliance on distance computation, which has a time complexity of O(n·d) where n is the number of training samples and d is the number of features. AdaBoost-SMOTE-ENN, with its ensemble-based structure, demonstrated faster inference, making it more appropriate for scenarios requiring real-time decision-making. Based on the runtime analysis, we suggest that AdaBoost-SMOTE-ENN is better suited for real-time applications due to its lower inference time and consistent predictive performance. The KNN–SMOTE-ENN model, while slightly slower during inference, remains a robust choice for offline or semi-real-time use cases.

- The research study also proposed a hybrid ensemble model on the ILPD and BUPA Liver Disorder Dataset. For the ILPD dataset, the model achieves an overall accuracy of 93.2%, whereas for the BUPA dataset, the model attains an accuracy of 95.4%. The Brier score loss for the ILPD dataset is 0.032 and 0.031 for the BUPA Liver Disorder Dataset. The ensemble learning method used in the provided code primarily employs a VotingClassifier, which combines predictions from multiple base classifiers (RandomForestClassifier and GradientBoostingClassifier).

Liver disease is becoming more and more common over time. There are multiple factors that are responsible for the same, such as unhealthy lifestyles, obesity, excessive consumption of alcohol, etc. Despite increased health consciousness, the introduction of idle lifestyles and luxuries continues to be prevalent. In these scenarios, this research study may prove to be quite useful to the world because early, timely, and precise prediction of liver disease plays an important role in increasing the life span of patients. This work has currently been done on a CSV file; we can further proceed to apply imaging techniques for liver disease detection, utilizing ultrasound and other liver diagnosis images. There are other advanced classifier algorithms and hybrid techniques that we can use, which will give us better accuracy. Application of Deep Learning models, such as Fuzzy Neural Network, CNN, and ANN (32) may also be performed to predict liver disease.

## Data Availability

The original contributions presented in the study are included in the article/supplementary material, further inquiries can be directed to the corresponding author.

## Footnotes

^1^ https://www.python.org

^2^ https://scikit-learn.org
